# Super-enhancer-driven core transcription factor FOXP1 delays endothelial cell senescence via phase separation-mediated SESN3 activation

**DOI:** 10.7150/thno.119709

**Published:** 2026-01-01

**Authors:** Lushuang Mao, Zhao-fu Liao, Dong Tang, Yumin Qiu, Min Yang, Yanshang Li, Yituan Xie, Weimin Feng, Ze-jun Zheng, Xiao-meng Liu, Jing-ru Ye, Shui-hong Lu, Xin-bin Tang, Ming Shi, Yun-fei Qu, Heng Li, Zhu-guo Wu, Shun Xu, Xinguang Liu, Junjun Ding, Jian-jun Xie, Jun Tao, Xing-dong Xiong

**Affiliations:** 1 Guangdong Provincial Key Laboratory of Medical Immunology and Molecular Diagnostics, The First Dongguan Affiliated Hospital, Guangdong Medical University, Dongguan 523808, P.R. China.; 2 Dongguan Key Laboratory of Aging and Anti-Aging, Institute of Aging Research, Guangdong Medical University, Dongguan, P.R. China.; 3 Department of Hypertension and Vascular Disease, The First Affiliated Hospital of Sun Yat-sen University, Guangzhou, P.R. China.; 4 Department of Biochemistry and Molecular Biology, Shantou University Medical College, Shantou 515041, P.R. China.; 5 Department of Neurosurgery, the Affiliated Huizhou First Hospital, Guangdong Medical University, Huizhou, Guangdong 516003, P.R. China.; 6 Institute of Biochemistry & Molecular Biology, School of Basic Medical Sciences, Guangdong Medical University, Dongguan, P.R. China.; 7 Cardiovascular Center, The First Dongguan Affiliated Hospital, Guangdong Medical University, Dongguan, P.R. China.; 8 Department of Cardiovascularology, Dongguan Tungwah Hospital, Dongguan, 523808, P.R. China.; 9 RNA Biomedical Institute, Sun Yat-Sen Memorial Hospital, Zhongshan School of Medicine, Sun Yat-Sen University, Guangzhou, P.R. China.

**Keywords:** epigenetic regulation, super-enhancer, FOXP1, liquid-liquid phase separation, endothelial cell senescence

## Abstract

**Rationale:** Endothelial cell senescence leads to endothelial dysfunction, thereby promoting the progression of atherosclerosis. Super-enhancers are crucial epigenetic cis-regulatory elements whose extensive reprogramming drives aberrant transcription in human diseases. However, the underlying mechanisms by which super-enhancers regulate endothelial cell senescence remain unclear. This study reveals the effect of liquid-liquid phase separation (LLPS) mediated by super-enhancer-driven core transcription factor FOXP1 on endothelial cell senescence.

**Methods:** The landscape of super-enhancers, chromatin accessibility, and transcriptome profiling were characterized during endothelial cell senescence by conducting CUT&Tag-seq with antibodies against H3K27ac, H3K4me1, and H3K4me3, along with assays for ATAC-seq and RNA-seq. The Coltron algorithm was used to identify core transcription factors in the process of endothelial cell senescence. Fluorescence recovery after photobleaching (FRAP), dCas9-KRAB CRISPRi, and the Optodroplet assay were utilized to confirm the phase separation properties of FOXP1. Functional experiments were employed to elucidate the effect of FOXP1 on endothelial cell senescence through LLPS.

**Results:** Senescent endothelial cells undergo significant changes in their epigenome. FOXP1 is identified as a core transcription factor, driven by super-enhancers, which delays endothelial cell senescence and inhibits atherosclerosis. Moreover, FOXP1 undergoes LLPS, which the 19 phase-forming amino acids within the intrinsically disordered region of FOXP1 are capable of maintaining its ability to delay endothelial cell senescence. Mechanistically, FOXP1 activates the target gene SESN3 and inhibits the mTORC1 signaling pathway through phase separation, a key event in delaying endothelial cell senescence. The clinical evidences support the potential role of FOXP1 and SESN3 as protective factors against atherosclerosis.

**Conclusion:** FOXP1 undergoes phase separation at its super-enhancer, recruiting transcription coactivators to form condensates. These condensates, in turn, facilitate binding with the SESN3 promoter and inhibit the mTORC1 signaling pathway, thereby delaying endothelial cell senescence.

## Introduction

Endothelial cell senescence contributes to endothelial dysfunction, a key event driving the progression of atherosclerotic plaques 1-3. The vascular endothelium functions as a systemically disseminated organ, executing distinct functions that involve complex mechanisms, including epigenetic alterations, senescence, inflammatory stimuli, mechanotransduction, metabolism, and other processes 1. Alteration of epigenetic information, which functions as a molecular switch for gene expression regulation, represents a reversible driver of the aging process 4, 5. Mammalian cell identity is maintained by H3K27ac-rich super-enhancers, a process that occurs prenatally 6, 7. Recent studies have shown that the intrinsically disordered regions (IDRs) of transcriptional coactivators form phase-separated condensates at these super-enhancers 8, 9. Within these condensates of liquid-liquid phase separation (LLPS), the transcriptional apparatus is compartmentalized, providing insights into the mechanisms that regulate key cell identity genes 10. In this context, elucidating the structures of LLPS condensates remains challenging, and effective intervention methods for targeting endothelial cell senescence are currently lacking.

Aberrant transcriptional events play a critical role in the pathogenesis and progression of diseases, typically governed by a group of core transcription factors regulated by super-enhancers 11. These core transcription factors not only regulate the expression of their own target genes but also bind to each other's super-enhancer regions to form core transcriptional regulatory circuitry (CRC) 12. As a member of the forkhead box (Fox) family of transcription factors, FOXP1 regulates cell growth, differentiation, angiogenesis, and cardiac development 13-15. Previous studies have indicated that FOXP1 regulates age-related processes, including mesenchymal stem cell senescence and ovarian aging 16, 17. Recently, increasing evidence has suggested that phase separation plays a crucial role in transcriptional regulation 18-20. However, it remains unclear whether FOXP1 undergoes liquid-liquid phase separation at super-enhancers, thereby regulating endothelial cell senescence.

In this study, we characterized the epigenomic landscape of endothelial cell senescence and found that the core transcription factor FOXP1 regulated by super-enhancers can delay endothelial cell senescence, alleviate endothelial dysfunction and atherosclerosis. FOXP1 forms LLPS condensates in which 19 key phase-forming amino acids located in the IDR1 region maintain the ability of FOXP1 to delay endothelial cell senescence. Additionally, FOXP1 activates the expression of the downstream target gene SESN3 through LLPS, thereby inhibiting the mTORC1 pathway. There is no doubt that phase separation represents a promising therapeutic target, which can significantly broaden drug development strategies for undruggable transcription factors. Critically, direct SESN3 activators can also serve as a novel clinical intervention approach. Our results highlight a novel epigenetic and phase separation mechanism in endothelial cell senescence and endothelial dysfunction, providing insights into potential targets for anti-atherosclerosis.

## Results

### Chromatin profiles were characterized during endothelial cell senescence

**Figure 1:**
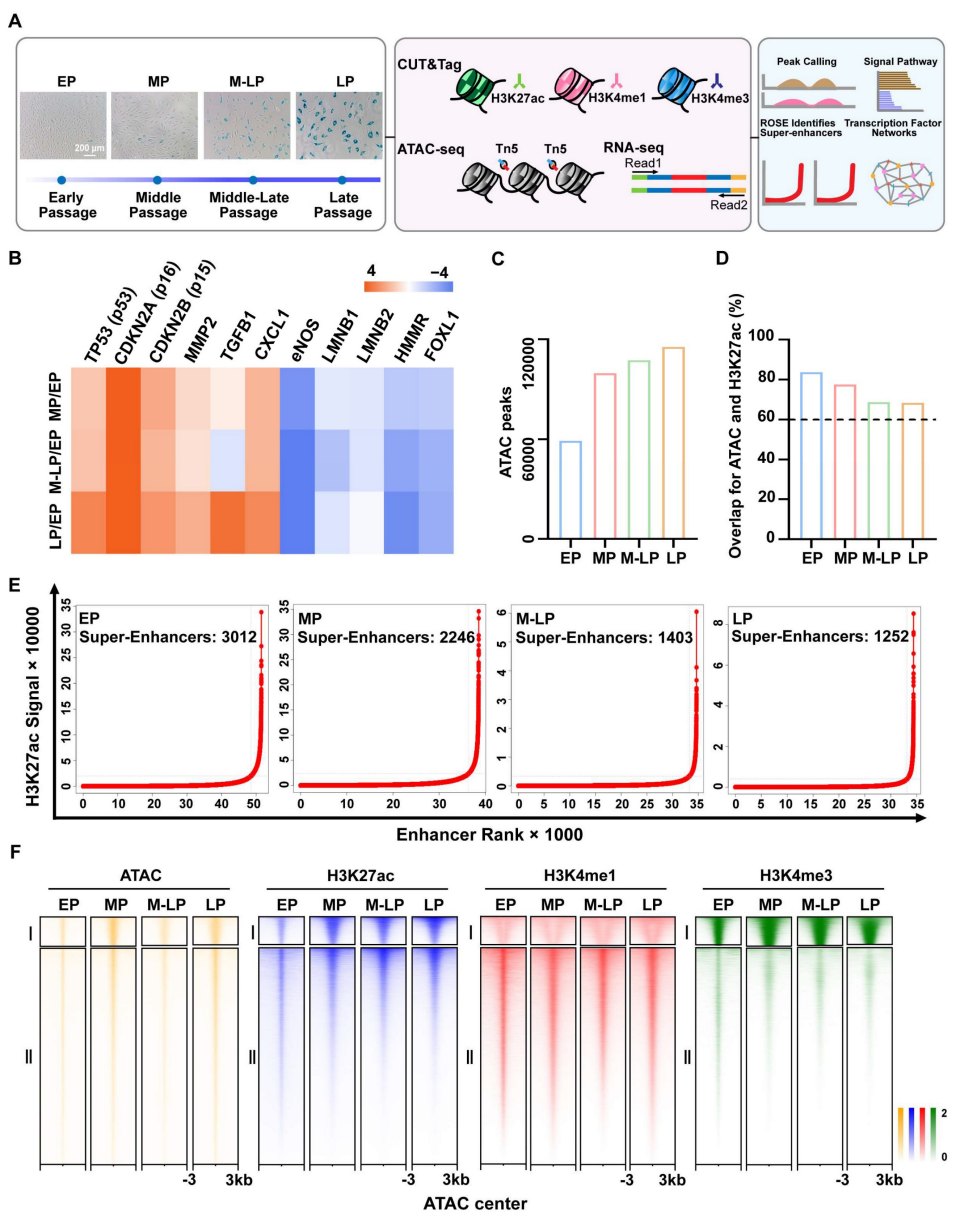
** Identification and characterization of super-enhancer profiles during endothelial cell senescence. (A)** Schematic diagram identifying epigenetic changes in early-passage (EP, P5, *n* = 3), middle-passage (MP, P15, *n* = 3), middle-late-passage (M-LP, P22, *n* = 3), and late-passage (LP, P30, *n* = 3) human umbilical vein endothelial cells. **(B)** Standardized RNA-seq data presented as a heatmap of senescence-related genes, colors represent fold change values after MP, M-LP, and LP were all compared to EP. **(C)** Total number of peaks in ATAC-seq. **(D)** Peak overlap rate between H3K27ac and ATAC-seq signal. **(E)** Enhancer-ranking hockey stick diagram defined by H3K27ac, enhancers above the inflection point of the curve have abnormally strong H3K27ac signals and are defined as super-enhancers. **(F)** ATAC-seq and CUT&Tag signals of either histone markers are grouped into two clusters by the k-means clustering algorithm at ± 3 kb windows around the center of ATAC peaks.

To characterize active cis-regulatory elements and accessible chromatin regions during endothelial cells senescence, we established a replicative senescence model by successive passaging of primary HUVECs. Subsequently, cells from four distinct stages were selected for RNA-seq, ATAC-seq (Assay for Transposase Accessible Chromatin with high-throughput sequencing) and CUT&Tag (Cleavage Under Targets and Tagmentation) sequencing of H3K27ac (active enhancers), H3K4me1 (active/equilibrium enhancers), H3K4me3 (active promoters). The stages were categorized as EP (Early Passage), MP (Middle Passage), M-LP (Middle-Late Passage), and LP (Late Passage). Late passage cells exhibited increased senescence-associated β-galactosidase (SA-β-gal) staining (Figure 1A), accompanied by reduced proliferative capacity and attenuated tubule formation (Figure S1A-B). The replicative senescent genes, including TP53, CDKN2A, and CDKN2B, exhibited elevated expression during senescence, whereas the endothelial dysfunction-associated gene eNOS showed a gradual decrease in expression (Figure 1B). As anticipated, the correlation of H3K27ac enrichment diminished progressively with the serial passaging of endothelial cells (Figure S1C).

A high degree of correlation was observed between ATAC-seq and histone-modified CUT&Tag enrichment, with peak overlaps between ATAC and H3K27ac exceeding 60% in all cases, thus confirming the important roles of histone modifications in chromatin accessibility (Figure 1D, Figure S2A). Furthermore, quality control analysis indicated that the insert fragment size distribution in ATAC-seq libraries was appropriate, that the ATAC signals following quantile normalization were displayed as box plots, and that the Q30 score for the ATAC, CUT&Tag, and RNA-seq reads was approximately 90%, with a mapping ratio to the human genome exceeding 75% (Figure S2B-F). Similar to ATAC occupancy, the H3K27ac and H3K4me1 peaks were predominantly located in intronic and intergenic regions, with a minor proportion at proximal promoters (Figure S1D). This was distinct from the H3K4me3 pattern, which exhibited the highest enrichment at promoter regions (Figure S1D). Super-enhancers are defined as clusters of enhancers with exceptionally high densities of H3K27ac, a hallmark of active gene expression that plays a crucial role in determining cell identity. Here, we observed that the number of super-enhancers in senescent cells exhibited a reduction (Figure 1E), while chromatin accessibility displayed an opposite trend (Figure 1C), suggesting that the epigenetic landscape undergoes dysregulation during the process of endothelial cell senescence. We identified all peaks across four stages, and the number of peaks observed in the genome indicates a more pronounced difference between the young and senescent stages of endothelial cells (Table S1). The accessible chromatin profiles were then clustered into two groups at the center of the ATAC peaks: (I) active promoters and (II) enhancers (Figure 1F). Overall, we established the chromatin landscape of endothelial cell senescence to facilitate further analysis.

### Super-enhancers undergo significant changes during vascular endothelial cell senescence

**Figure 2:**
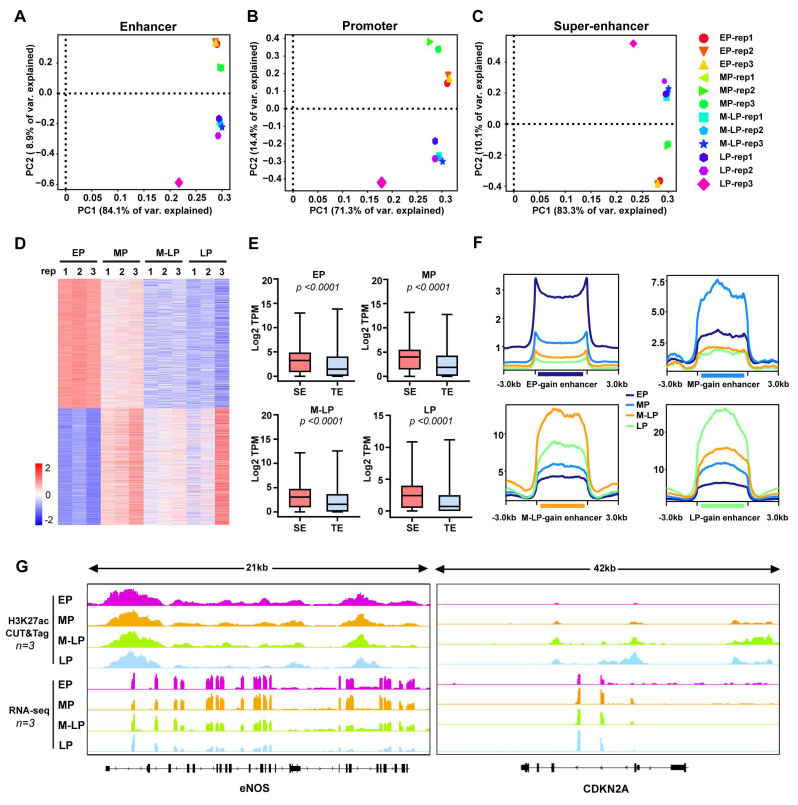
** The H3K27ac profiles define the active regulatory elements during endothelial cell senescence. (A-C)** PCA of enhancers, promoters, and super-enhancers across four stages of endothelial cells. **(D)** Differential peak analysis of H3K27ac enrichment signals. **(E)** Normalized expression of each generation in SE and TE-related genes, SE: super-enhancer, TE: typical enhancer. *P* value was calculated using an unpaired *t*-test. **(F)** A comparison of H3K27ac signal enrichment in gained enhancers is conducted using EP gain/loss enhancers as an example to illustrate the selection criteria: the peaks from the remaining three generations were combined, and the H3K27ac signal values of EP and the combined peaks from the other three generations were compared using DESeq2; a log2 fold change > 1 indicates gain, while a log2 fold change < -1 indicates loss. **(G)** Viewable H3K27ac CUT&Tag signal and RNA-seq at the* eNOS* and *CDKN2A* gene loci.

To explore altered patterns of epigenomic super-enhancers, we first identified promoter, enhancer, and super-enhancer elements across the four stages of endothelial cells described above. The differential promoter-, enhancer- and super-enhancer-associated genes were subjected to principal component analysis (PCA), which successfully separated into group-specific traits (Figure 2A-C). Next, the differential super-enhancers were identified based on the signal density of H3K27ac, with the peaks showing increased divergence as senescence progressed (Figure 2D). Importantly, genes associated with super-enhancers were expressed at significantly higher levels compared to typical enhancers (Figure 2E). In accordance with previous studies, we defined gained or lost enhancers and super-enhancer as those with an absolute value of the fold change (FC) of the H3K27ac reads per kilobase per million reads (RPKM) was ≥ 2 , an absolute difference ≥ 0.5 21, 22. Subsequently, these differential enhancers were classified into eight groups: E1 (EP gain), E2 (EP loss), E3 (MP gain), E4 (MP loss), E5 (M-LP gain), E6 (M-LP loss), E7 (LP gain), E8 (LP loss) (Figure S3A-B). As expected, the gained enhancer of each stage has the highest H3K27ac signal density (Figure 2F). Genes directly regulated by gained or lost super-enhancers were identified using HOMER. Interestingly, genes regulated by gained enhancers in young cells, such as endothelial nitric oxide synthase (eNOS), exhibit higher expression levels, while CDKN2A displays the opposite pattern (Figure 2G). Furthermore, we analyzed the differences in chromatin accessibility across the four stages (Figure S3C), noting that more peaks were shared between EP and MP than those between EP and M-LP or LP (Figure S3D). This finding indicates that the genome underwent significant divergence at the senescent stage, exhibiting a greater degree of differentiation in regulatory elements compared to the proliferating stages. The gene expression levels were positively correlated with its chromatin accessibility during endothelial cell senescence (Figure S3E).

Given the key roles of enhancers and super-enhancers in the process of transcriptional activation, we compared the distinct four stages of endothelial cells in pairs to identify gained or lost super-enhancers and enhancers based on the aforementioned criteria (Figure S4A-D). The integration of RNA-seq data from pairwise comparisons of four stages of cells reveals that the expression levels of genes associated with gained or lost super-enhancers and enhancers were up-regulated or down-regulated accordingly. Changes were more pronounced in comparisons between stages with greater age difference (e.g, LP vs. EP) than in those between stages of similar age (e.g, MP vs. EP) (Figure S4B and D). These results suggest that the epigenetic remodeling associated with aging exhibits characteristics of phased accumulation. Gene ontology (GO) pathway analysis revealed that enhancer-related genes gained in M-LP and LP were enriched in fundamental processes of senescence, including negative regulation of endothelial cell proliferation and migration, response to hypoxia, and TGF-β signaling pathway. In contrast, loss of super-enhancer in MP, M-LP, and LP were enriched in cellular response to oxidative and chemical stress, and regulation of Wnt signaling pathway (Figure S5A-F). The above results suggest that differential super-enhancers in senescent endothelial cells are associated with an aberrant transcriptional programme.

### The regulatory network formed by core transcription factors was characterized during the process of endothelial cell senescence

**Figure 3:**
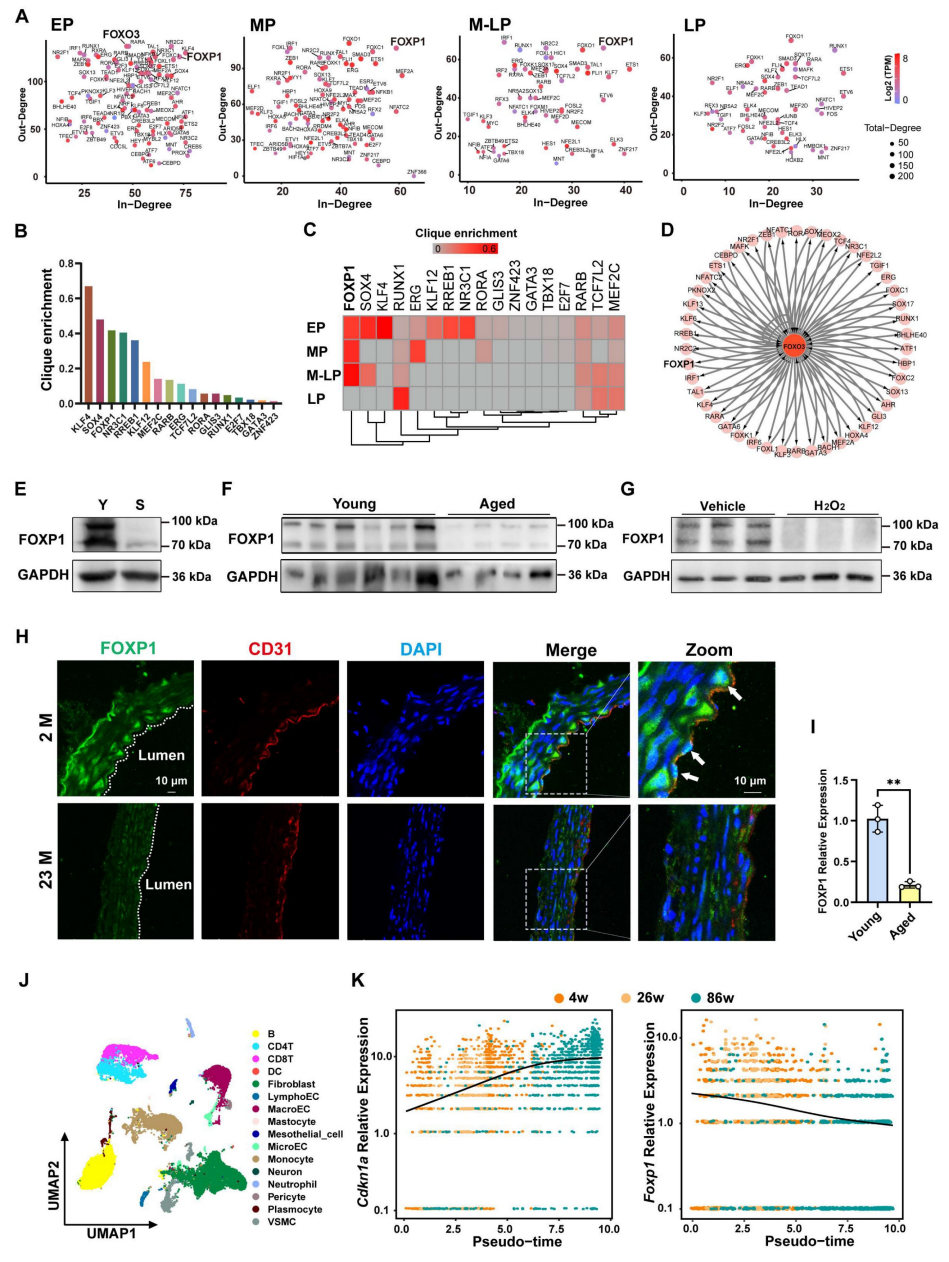
** The core transcription factor FOXP1 is driven by super-enhancer in young endothelial cells. (A)** Scatter plot of In-degree (inward degree, the number of TFs binding to the super-enhancer of a node TF) and Out-degree (outward degree, number of super-enhancers bound by a node TF) of the TFs driven by super-enhancer from EP to LP. **(B)** Clique enrichment score of each CRC TF calculated as the percentage of total cliques in which that TF is a constituent member in EP. **(C)** Candidate core transcription factors ranked by clique enrichment across four stages of cells. **(D)** A FOXO3-centered network in EP showing super-enhancer-based regulatory relationships. **(E)** Western blot analysis of FOXP1 expression in young and senescent HUVECs. Y: young, S: senescent. **(F)** Western blot analysis of FOXP1 expression in the aortas of young (*n* = 6) and old (*n* = 4) mice. **(G)** Western blot analysis of FOXP1 expression in HUVECs treated with 100 μM H_2_O_2_ (*n* = 3). **(H)** Immunofluorescence staining of FOXP1 in the aortas of young and old mice. 2M: 2 months, 23M: 23 months (*n* = 3 mice per group). **(I)** RT-qPCR analysis of FOXP1 expression in the intima of young and aged mice (*n* = 3 mice per group). **(J)** Clustering of different cell types in mice arteries. **(K)** Changes in Cdkn1a and Foxp1 expression levels along the pseudo-time trajectory. Data are presented as means ± SD, Student's *t*-test, **P < 0.01.

It has been demonstrated that aberrant transcriptional events play a pivotal role in the development of diseases 23. To construct the core transcriptional regulatory circuitry during the process of endothelial cell senescence, the Coltron package was applied to the H3K27ac CUT&Tag data described above, identifying the core transcription factors (Figure 3A). As senescence progresses, the number of core transcription factors gradually decreases, indicating a significant change in transcriptional events (Figure 3A). Particularly, FOXO3, a classical longevity gene, has been shown that dysregulation of FOXO3 contributes to a variety of vascular aging-related diseases 24-27. It was identified as a core transcription factor specifically in EP (young endothelial cells), but not in other cell generations (Figure 3A). To identify the transcription factor that delays endothelial cell senescence, the genes annotated with the gained enhancers of EP were intersected with the core transcription factors of EP, sorting out 17 candidate transcription factors with clique enrichment (Figure 3B, table S2). Interestingly, FOXP1 exhibited the most significant difference in the enrichment of the CRC clique between the young and the senescent stages (Figure 3C). In the regulatory network with FOXO3 as the central node, FOXP1 exhibits a mutual regulatory relationship with FOXO3 (as indicated by double arrows) (Figure 3D). Our RNA-seq data illustrate changes in the expression of candidate transcription factors across four stages (Figure S6A). As anticipated, the expression of FOXP1 is down-regulated in senescent endothelial cells and the aortas of aged mice (Figure 3E-F). Consistently, FOXP1 levels are similarly reduced upon H₂O₂-induced oxidative stress senescence (Figure 3G). Importantly, FOXP1 exhibited low expression in the intima of aged mice (Figure 3H-I). Subsequently, by analyzing published single-cell data from the aortas of 4-, 26-, and 86-week-old mice, we found that macroendotheliocytes (MacroECs) exhibit higher gene set activity related to cellular senescence 28. Thus, we conducted pseudotime trajectory analysis on MacroECs and observed the upregulation of Cdkn1a and the downregulation of Foxp1 as age increased (Figure 3J-K). Furthermore, we analyzed the human carotid atheroma RNA-seq data (GEO: GSE43292), comprising 32 DMIT (distant macroscopically intact tissues) and 32 CP (core of the plaque) samples. The expression of FOXP1 in DMIT was found to be higher than in CP (Figure S6B). These results indicate that FOXP1 may function as a pivotal transcription factor associated with endothelial cell senescence and atherosclerosis.

FOXP1 expression was down-regulated in endothelial cells upon treatment with JQ1 and THZ1 (Figure S6C), inhibitors of BRD4 and CDK7 (components of the super-enhancer complex) 29, 30. Epigenomic profiling revealed robust co-enrichment of H3K27ac, ATAC-seq accessibility, and H3K4me3 signals at a predicted super-enhancer region around the FOXP1 gene locus (Figure S6D). To functionally validate the activity of the predicted FOXP1 super-enhancer, its constituent enhancer elements were cloned individually into pGL3-promoter luciferase reporter vector and then transfected into HEK293T cells. Robust reporter activities were observed for all tested enhancer components, consistent with the high enrichments of H3K27ac and H3K4me1 in these regions (Figure S6E). Next, we specifically targeted the three most active enhancer elements (E3-E5) using the dCas9-KRAB CRISPRi method, which combines a deactivated Cas9 (dCas9) with the Krüppel-associated box (KRAB) repressor domain to selectively inhibit gene expression by binding to specific DNA sequences 31, 32. Western blot and RT-qPCR analyses showed that single guide RNAs (sgRNAs) targeted these enhancers significantly reduced FOXP1 expression (Figure S6F). Taken together, these results suggest that FOXP1 is transcriptionally activated by a super-enhancer, which is responsible for its high-expression in the early stage of endothelial cell.

### FOXP1 delays endothelial cell senescence, alleviates endothelial dysfunction and atherosclerosis

**Figure 4:**
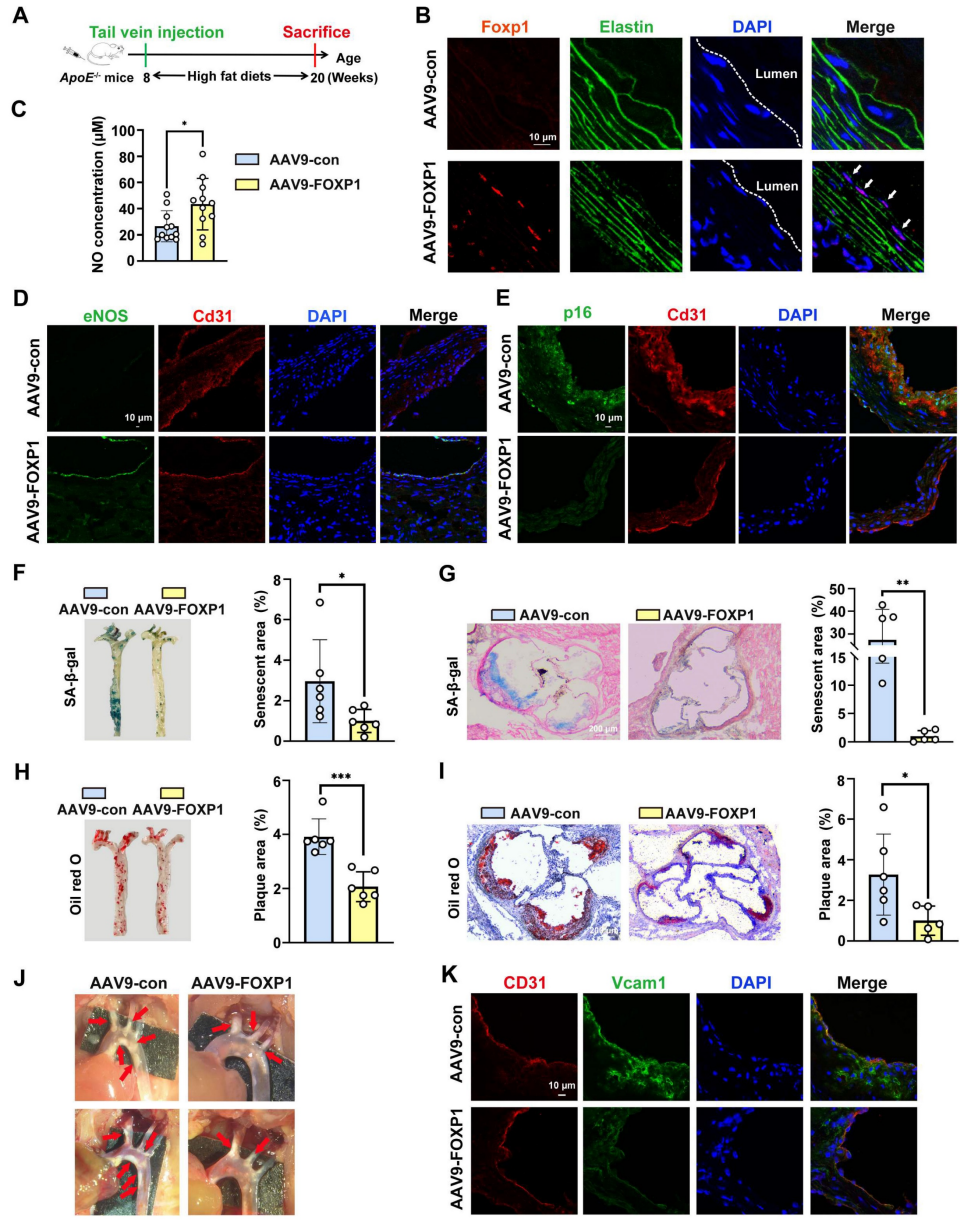
** Endothelial cell-specific overexpression of FOXP1 can delay endothelial cell senescence, alleviate endothelial dysfunction and atherosclerosis. (A)** Schematic diagram of the time nodes of tail vein injection of AAV9-con and AAV9-FOXP1. **(B)** Immunofluorescence staining of FOXP1 in the intima of ApoE KO mice following injection of AAV9-Con or AAV9-FOXP1. **(C)** Colorimetric detection of serum NO concentration in mice endothelial cell-specific overexpression AAV9-FOXP1 group or AAV9-Con group (*n* = 11 mice per group). **(D-E)** Immunofluorescence staining of eNOS and p16 in the intima of ApoE KO mice following injection of AAV9-Con or AAV9-FOXP1. **(F-G)** SA-β-gal staining of whole aorta and aortic roots of ApoE KO mice following injection of AAV9-Con or AAV9-FOXP1. **(H-I)** Oil red O staining of whole aorta and aortic roots of ApoE KO mice following injection of AAV9-Con or AAV9-FOXP1. **(J)** Plaques in the aorta arches of ApoE KO mice following injection of AAV9-Con or AAV9-FOXP1. *n* = 6 mice per group. **(K)** Immunofluorescence staining of Vcam1 and CD31 in the intima of ApoE KO mice following injection of AAV9-Con or AAV9-FOXP1. Data are presented as means ± SD, Student's *t*-test, *P < 0.05, **P < 0.01, ***P < 0.001.

To further investigate the function of FOXP1, FOXP1 was knocked down and overexpressed in endothelial cells using siRNA and lentivirus, respectively. Interestingly, the downregulation of FOXP1 using either the siRNA or the dCas9-KRAB-sgRNA CRISPRi system significantly promotes cellular senescence, while simultaneously inducing hyperpermeability, inhibiting tube formation, and suppressing cell proliferation (Figure S7A-J). Conversely, the overexpression of FOXP1 exhibits an opposite trend (Figure S7K-O), suggesting that FOXP1 can delay vascular endothelial cell senescence and alleviate endothelial dysfunction. To elucidate the impact of FOXP1 on vascular endothelial senescence and atherosclerosis *in vivo*, ApoE knock-out (KO) mice were injected with recombinant adeno-associated virus 9 (AAV9) carrying FOXP1 under the control of an endothelial-specific promoter ICAM2 (ICAM2-AAV9-FOXP1) (Figure 4A). Aortas were sampled after a 12-week period of a high-fat diet. Immunofluorescence staining was conducted to confirm the successful delivery of FOXP1 in endothelial cells (Figure 4B). A comparison was made of the nitric oxide (NO, a marker of endothelial dysfunction) 33 concentration in the serum, and treatment with AAV9-FOXP1 significantly increased NO levels compared to AAV-con (Figure 4C). Consistently, the AAV9-FOXP1 treatment exhibited a greater expression of eNOS than the AAV9-con (Figure 4D). These results suggest that FOXP1 has a significant effect in alleviating endothelial dysfunction. Moreover, the AAV9-FOXP1 treatment reduced the expression of p16, p21, and p53 in the intima compared to the AAV9-con (Figure 4E, Figure S8A-B). In addition, *en face* analyses of atherosclerotic senescent and plaque areas in the whole aortas and the aortic roots revealed that FOXP1 protected against endothelial senescence and atherosclerosis (Figure 4F-I). Endothelial-specific overexpression of FOXP1 inhibited atherosclerotic plaque formation (Figure 4J). Importantly, treatment with AAV9-FOXP1 showed lower expression of Vcam1 and Icam1 compared to AAV9-con (Figure 4K). These results suggest that FOXP1 can delay endothelial cell senescence, alleviate endothelial dysfunction and atherosclerosis.

### FOXP1 condensation at the super-enhancer region exhibits phase separation properties

**Figure 5:**
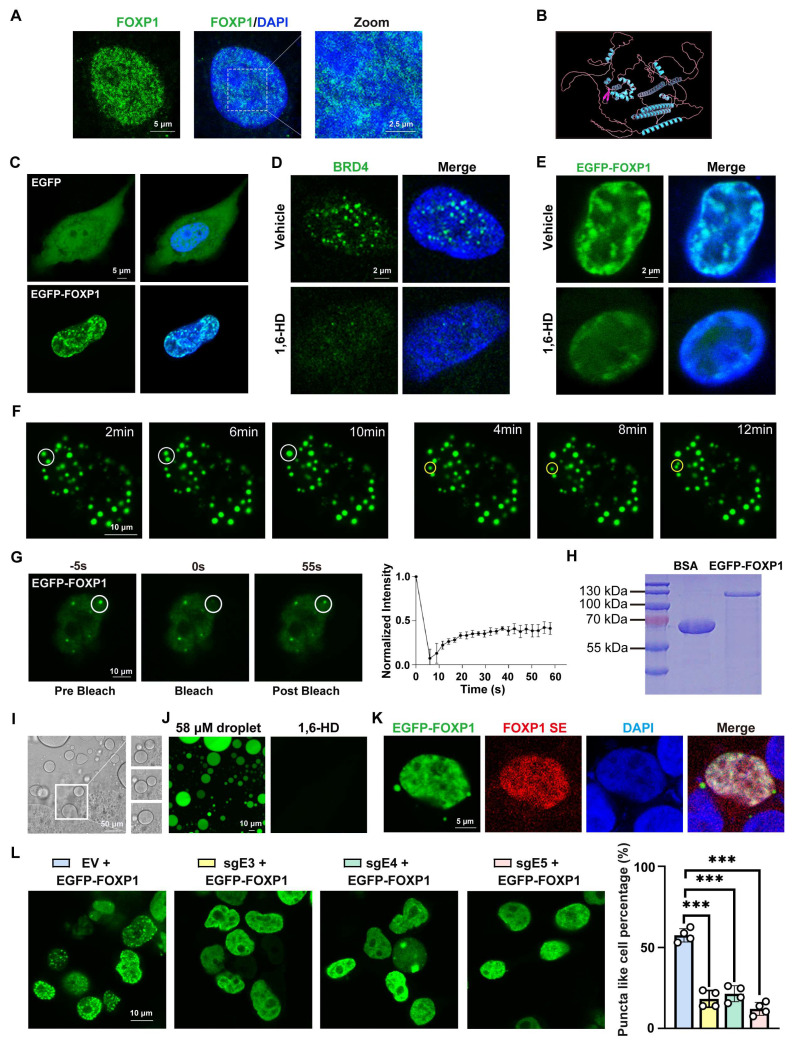
** FOXP1 forms condensates at its super-enhancer. (A)** Immunofluorescence staining for FOXP1 in HUVECs. **(B)** Schematic representation of the three-dimensional structure of the FOXP1 protein. **(C)** Phase separation was observed after transient transfection of pcDNA3.1-EGFP-FOXP1 plasmid into HUVECs-SV40. **(D)** 10% 1,6-HD treatment for 5 min resulted in the disappearance of endogenous BRD4 condensates. **(E)** The disappearance of FOXP1 phase separation after treatment with 10% 1,6-HD for 5 minutes in HUVECs-SV40. **(F)** Time-lapse imaging to record the fusion (white circle) and fission (yellow circle) of EGFP-FOXP1 protein droplets occurring over time. **(G)** Images of fluorescence recovery of HUVECs-SV40 expressing EGFP-FOXP1 recorded by FRAP assay (left), quantification of fluorescence intensity during FRAP assay (right). **(H)** Coomassie Brilliant Blue staining of purified recombinant EGFP-FOXP1 proteins after being resolved on SDS-PAGE. **(I)** Time-lapse DIC microscopy captures spontaneous fusion events between EGFP-FOXP1 droplets. **(J)** Disappearance of EGFP-FOXP1 droplets after 10% 1,6-HD treatment for 5 min. **(K)** To identify the colocalization between FOXP1's super-enhancer and FOXP1 condensates, the probes for DNA-FISH were designed at FOXP1's super-enhancer region, EGFP-FOXP1 plasmid were transfected into HEK293T cells to form condensates. **(L)** Phase separation of FOXP1 was observed in HEK293T cells co-transfected with dCas9-KRAB super-enhancer and EGFP-FOXP1 plasmids, *n* = 4 biological replicates. Data are presented as means ± SD, one-way ANOVA, ***P < 0.001.

Recent studies have indicated that many TFs may form phase-separated condensates to exert their transcriptional regulatory effects 34-36. Therefore, research was conducted to determine whether FOXP1, as a master transcription factor, could also undergo phase separation. In our study, we first examined the subcellular distribution of endogenous FOXP1 and observed the formation of nuclear puncta in endothelial cells (Figure 5A), suggesting its potential capacity for phase separation. IDR-containing proteins constitute a significant class of macromolecules that exhibit physiological phase separation 37, 38. AlphaFold structural prediction revealed that FOXP1 contains extensive IDRs, accounting for approximately 70% of its primary sequence (Figure 5B).

Next, an enhanced green fluorescent protein (EGFP)-FOXP1 fusion protein was expressed in immortalized human umbilical cord vein endothelial cells (HUVECs-SV40) and HEK293T cells. As predicted, FOXP1 exhibited droplet-like puncta in the nucleus, in contrast to the uniform and diffuse distribution of EGFP throughout the cell (Figure 5C, Figure S9A). It has been demonstrated that BRD4, a phase-separated protein, is susceptible to disruption by 1,6-hexanediol (1,6-HD), a chemical compound known to disrupt condensates 39. Treatment of BRD4 with 1,6-HD resulted in the dissolution of the BRD4 droplets (Figure 5D). A similar response was observed with FOXP1 droplets, which exhibited LLPS properties (Figure 5E). Moreover, time-lapse imaging revealed that EGFP-FOXP1 droplets underwent spontaneous fusion and fission (Figure 5F, Movie 1). Fluorescence recovery after photobleaching (FRAP) assays further verified the rapid exchange kinetics of FOXP1 droplets (Figure 5G, Movie 2). These findings strongly suggest that the FOXP1 protein possesses LLPS properties in vascular endothelial cells. For *in vitro* validation, we purified full-length EGFP-FOXP1 protein from *E. coli* (Figure 5H). The recombinant EGFP-FOXP1 protein spontaneously formed dynamic droplets that underwent fusion over time (Figure 5I, Movie 3) and these droplets were could also be disrupted by 1,6-HD (Figure 5J). Single-molecule fluorescence *in situ* hybridization (FISH) coupled with EGFP-FOXP1 fusion expression plasmid revealed spatial colocalization of FOXP1 condensates with its super-enhancer (Figure 5K). The use of dCas9-KRAB CRISPRi to mediate the suppression of super-enhancer activity through sgRNAs resulted in the disruption of FOXP1 LLPS (Figure 5L), suggesting that FOXP1 forms condensates at super-enhancers.

### FOXP1 delays endothelial cell senescence through IDR1-dependent LLPS

**Figure 6:**
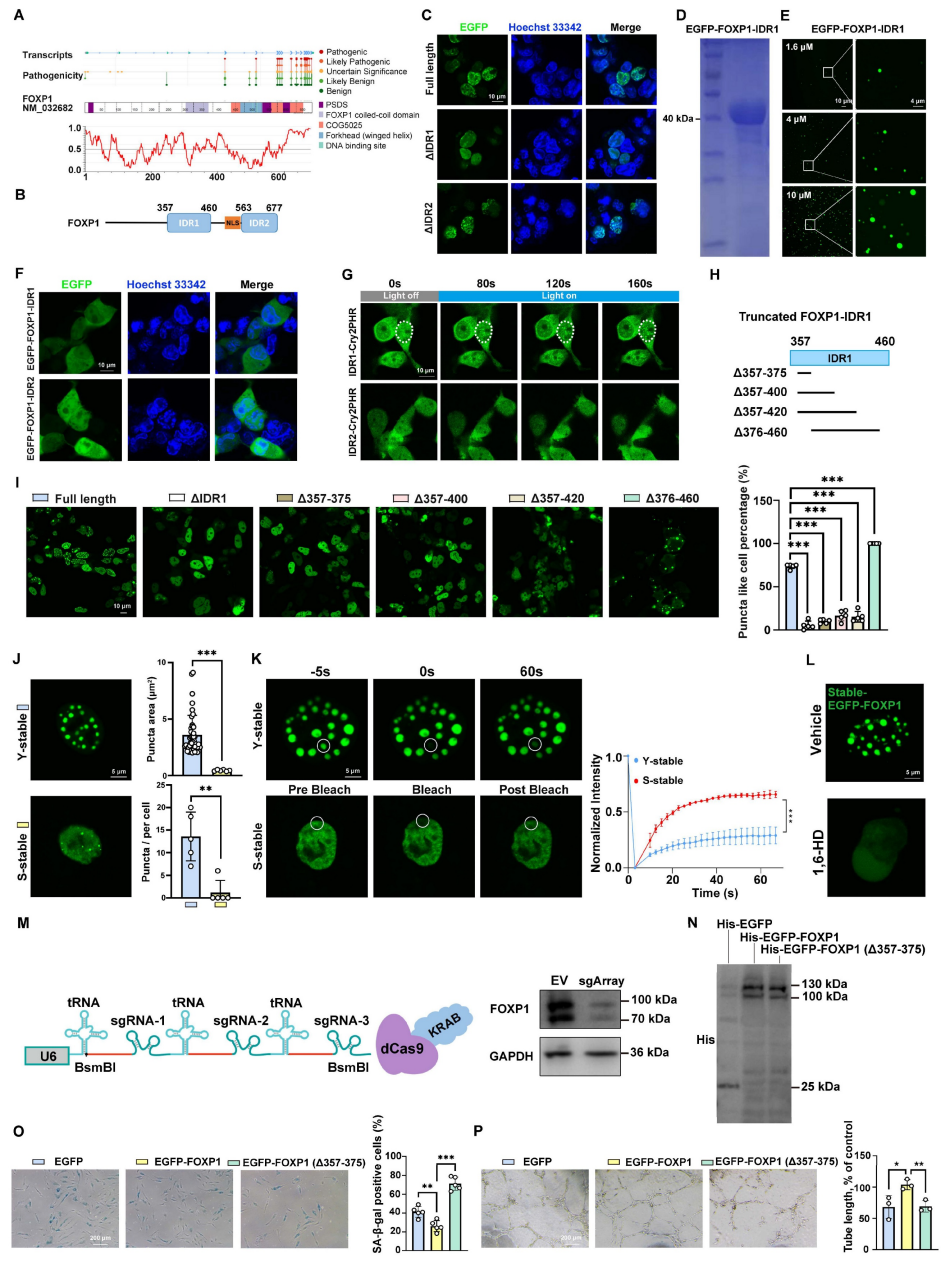
** FOXP1 exhibits enhanced phase-separated condensate formation in young endothelial cells. (A)** Disease-associated mutations mapped onto FOXP1 and disorder as IUPred2A score. **(B)** Schematic representation of the IDR region of FOXP1. **(C)** Comparative analysis of condensate formation in HEK293T cells expressing full-length EGFP-FOXP1 versus those with deletions of IDR1 or IDR2. **(D)** Coomassie Brilliant Blue staining of purified recombinant EGFP-FOXP1-IDR1 proteins resolved by SDS-PAGE. **(E)** Images of the density and number of droplets of EGFP-FOXP1-IDR1 proteins in different concentrations. **(F)** The transfection of EGFP-FOXP1-IDR1 or EGFP-FOXP1-IDR2 fragments into HEK293T cells did not exhibit LLPS. **(G)** The optoDroplet assay was conducted with time-lapse imaging at a wavelength of 488 nm, following the fused expression of the blue photoreceptor protein Cry2PHR with either IDR1 or IDR2. **(H)** Schematic of IDR1 truncation strategy. **(I)** Phase separation was observed after truncation of IDR1; at least 100 HEK293T cells were counted. **(J)** Representative confocal images of FOXP1 condensates in young and senescent HUVECs stably expressing EGFP-FOXP1. **(K)** FRAP analysis of EGFP-FOXP1 condensates in young and senescent HUVECs. Time-lapse images (left); quantification of fluorescence intensity during FRAP assay (right). **(L)** Condensates that stably expressed EGFP-FOXP1 were disrupted by 1,6-hexanediol. **(M)** Multiplexed CRISPRi design: Three sgRNAs were designed in the promoter region of FOXP1 and ligated to dCas9-KRAB vector using tRNA sequences in tandem, which were packaged into lentivirus and then infected with HUVECs. **(N)** Western blot analysis for His in HUVECs transduced with lentiviruses expressing His-EGFP, His-EGFP-FOXP1 and His-EGFP-FOXP1 (Δ357-375). **(O-P)** SA-β-gal staining (O) and tube formation assay (P) in HUVECs with dCas9-KRAB CRISPRi-mediated knockdown of endogenous FOXP1 followed by overexpression of EGFP, EGFP-FOXP1 and EGFP-FOXP1 (Δ357-375) variants, respectively. Data are presented as means ± SD, one-way ANOVA in I, O and P; Student's *t*-test in K, *P < 0.05, **P < 0.01, ***P < 0.001.

Recent studies have indicated that the low complexity of IDRs contributes to LLPS 40, 41. Utilizing IUPred2A 42, two potential IDR regions of FOXP1 were identified: IDR1 (357-460 aa) and IDR2 (563-677 aa) (Figure 6A-B). To assess the importance of two IDRs, expressing truncation variants of FOXP1 revealed that the deletion of IDR1 abolished FOXP1 LLPS in cells, whereas IDR2 did not (Figure 6C). To further explore its role, the recombinant EGFP-FOXP1-IDR1 protein was purified (Figure 6D), and the droplet formation assay was performed with varying concentrations (from 1.6 to 10 μM). EGFP-FOXP1-IDR1 formed spherical droplets in a concentration-dependent manner (Figure 6E). EGFP-FOXP1-IDR1 droplets were disrupted by 1,6-hexanediol (Figure S9B) and exhibited rapid FRAP recovery (Figure S9C). Since phase separation proteins are typically highly responsive to changes in environmental factors such as salt concentration and molecular crowding, it has been demonstrated that PEG 4000 promotes phase separation, whereas high concentrations of sodium chloride inhibit this process (Figure S9D-E). Moreover, the turbidity of the EGFP-FOXP1-IDR1 protein solution at the same concentration was observed to gradually increase with the addition of PEG 4000 (Figure S9F). The optical density (OD) of the EGFP-FOXP1-IDR1 protein solution at 600 nm (OD600) was measured using a UV spectrophotometer. And it was observed that the OD600 increased in direct proportion to the concentration of PEG 4000 (Figure S9F). Droplet fusion experiments revealed that the EGFP-FOXP1-IDR1 droplets separated for a short time and then fused again (Figure S9G). Furthermore, some droplets exhibit the characteristics of a spherical shell (Figure S9H). Collectively, these results establish FOXP1 as a phase-separating protein both *in vitro* and *in vivo*, with the IDR1 domains being essential for FOXP1 LLPS.

Intriguingly, neither isolated IDR1 nor IDR2 exhibited autonomous condensation (Figure 6F). OptoDroplet assays revealed the inducibility of blue light on IDR1-Cry2PHR condensates, but not on IDR2-Cry2PHR (Figure 6G). Additionally, the LLPS deficiency caused by the deletion of IDR1 can be rescued through fusion with phase-separating domains, such as those found in FUS or hnRNPA1 IDR (Figure S10). To identify the minimal sequences of key phase-separation residues, we followed the strategy of truncated IDR1 (Figure 6H). The fine truncations of sequences from 357-375 aa, 357-400 aa, and 357-420 aa abolished puncta formation, strikingly. The removal of 376-460 aa led to ectopic cytoplasmic condensation (Figure 6I), indicating a context-dependent manner of phase separation. Therefore, 357-375 aa is confirmed to be essential for the liquid-liquid phase separation of FOXP1.

To elucidate the relationship between FOXP1-mediated LLPS and endothelial cell senescence, the EGFP-FOXP1 fusion plasmid was packaged into lentiviral particles and subsequently used to transduce both young and senescent endothelial cells. Live-cell imaging revealed larger and more numerous FOXP1 droplets in young endothelial cells (Figure 6J), and the FRAP assay showed a slower rate of fluorescence recovery in young endothelial cells compared to senescent endothelial cells (Figure 6K), suggesting that the phase separation of FOXP1 may be involved in the regulation of endothelial cell senescence. 1,6-hexanediol performs the same function of disrupting LLPS in the endothelial cells stably expressing EGFP-FOXP1 (Figure 6L). Considering the fact that phase separation differs between young and senescent endothelial cells, we further investigated the impact of disrupting FOXP1 phase separation on cellular senescence.

Endogenous FOXP1 expression was knocked down in endothelial cells using the dCas9-KRAB CRISPRi system (Figure 6M). Subsequently, the wild-type FOXP1 plasmid (FOXP1), the truncated FOXP1 plasmid (FOXP1-Δ357-375), and the empty vector (EGFP) were overexpressed, respectively. Each of these plasmids was fused to express EGFP and His tags (Figure 6N). SA-β-gal staining and tube formation assays indicated that overexpression of FOXP1 significantly delayed endothelial senescence and alleviated endothelial dysfunction. Conversely, deletion of the 19 phase-separation key residues diminished this effect (Figure 6O-P). In conclusion, these results emphasize the significant role of FOXP1 phase separation in delaying endothelial cell senescence.

### FOXP1-mediated LLPS delays endothelial cell senescence by activating SESN3 expression

**Figure 7:**
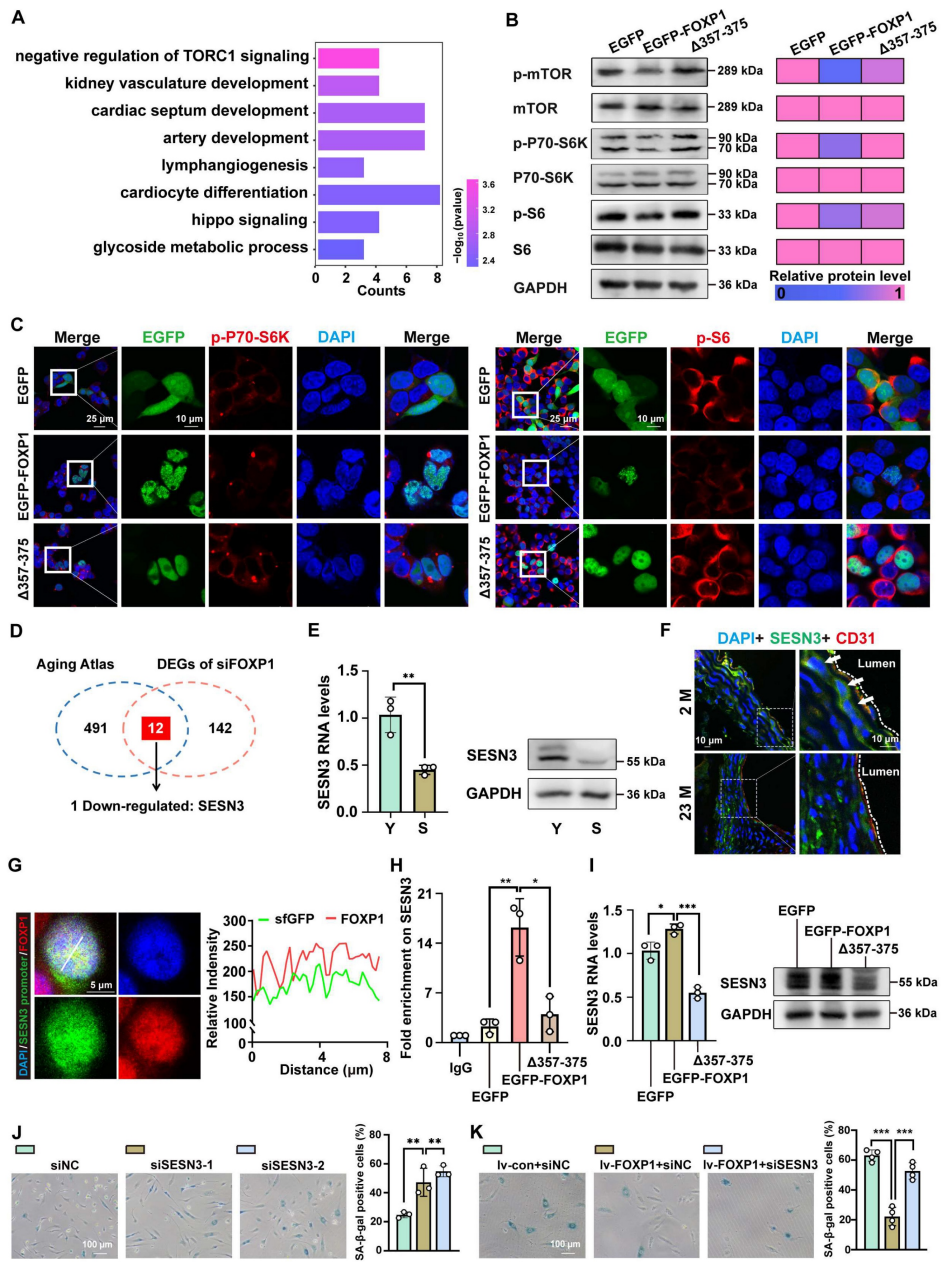
** FOXP1 LLPS drives SESN3 transcriptional activation and then inhibits mTORC1 signaling. (A)** GO enrichment analysis was conducted for DEGs following siFOXP1 treatment. **(B)** Western blot assay to evaluate the impact of FOXP1 LLPS on the phosphorylated forms of mTOR, S6, and P70-S6K in HUVECs. **(C)** Immunofluorescence assay for the effect of FOXP1 LLPS on p-S6 and p-P70-S6K in HEK293T cells. **(D)** The venn diagram indicates that 154 DEGs after FOXP1 knockdown intersect with 503 known senescent-related genes in the Aging Atlas. **(E)** RNA and protein levels of SESN3 were examined in young and senescent HUVECs. **(F)** Immunofluorescence staining of SESN3 in the aortas of young and aged mice. 2M: 2 months, 23M: 23 months. **(G)** The SESN3 promoter was visualized using the dCas9-SunTag system and analyzed for colocalization with endogenous FOXP1 in HEK293T cells. **(H)** CUT&Tag-qPCR assays for the ability of EGFP, EGFP-FOXP1 and EGFP-FOXP1 (Δ357-375) variants to bind to the SESN3 promoter region. **(I)** RNA and protein levels were examined to detect the effect on SESN3 after disruption of FOXP1 LLPS in HUVECs. **(J)** SA-β-Gal staining of HUVECs after knockdown of SESN3. **(K)** SA-β-Gal staining of HUVECs transduced with lentivirus overexpressing FOXP1 followed by knockdown of SESN3 using siRNA. Data are presented as means ± SD, one-way ANOVA in H, I, J and K; Student's *t*-test in E, *P < 0.05, **P < 0.01, ***P < 0.001.

Considering the established role of LLPS in the function of FOXP1, particularly in relation to endothelial senescence, this study sought to investigate the potential of FOXP1 phase separation as a regulatory mechanism. Among the differentially expressed genes (DEGs) after FOXP1 knockdown, the mTORC1 pathway was significantly enriched (Figure 7A). To determine whether FOXP1 phase separation mechanistically inhibits mTORC1 signaling, we quantified the phosphorylation levels of mTOR and its downstream effectors (p70S6K and S6) across EGFP, EGFP-FOXP1, and EGFP-FOXP1 (Δ357-375) variants. Both Western blot and immunofluorescence results confirmed that the disruption of FOXP1 LLPS activates the mTORC1 signaling pathway (Figure 7B-C). Integrative analysis of the Aging Atlas database 43 and DEGs upon FOXP1 knockdown revealed that 12 genes were enriched. Among these, SESN3 emerged as the sole target gene requiring regulation by FOXP1 (Figure 7D). Recent studies have provided compelling evidence indicating that SESN3 is a repressor of mTORC1 44. Similar to FOXP1, SESN3 was downregulated in senescent endothelial cells (Figure 7E). Importantly, SESN3 exhibited low expression in the intima of aged mice (Figure 7F). The dCas9-SunTag sgARRAY-mediated *in situ* labeling revealed that endogenous FOXP1 condensates colocalized with the SESN3 promoter in cells (Figure 7G). Through CUT&Tag-qPCR analysis of His antibody, we confirmed that FOXP1 highly occupies the SESN3 promoter region, while FOXP1 (Δ357-375) demonstrates a significant reduction in binding to this locus (Figure 7H). Notably, our data suggested that FOXP1-mediated LLPS elevates SESN3 expression (Figure 7I). The knockdown of SESN3 resulted in an increase in cellular senescence among endothelial cells (Figure 7J). Consistently, the knockdown of SESN3 can reverse the senescent phenotype induced by the overexpression of FOXP1 (Figure 7K). Moreover, FOXP1 condensates colocalize with other epigenetic regulators, such as RNA polymerase II, BRD4, H3K4me1, and others, indicating the complex interplay among FOXP1 LLPS, epigenetic states, and gene transcription, emphasizing their closely interconnected regulatory mechanisms (Figure S11). Overall, these findings revealed that FOXP1 forms condensates with transcriptional coactivators and subsequently increases the expression of SESN3, an inhibitor of the mTORC1 signaling pathway, thereby delaying endothelial cell senescence.

### FOXP1 and SESN3 are potential protective factors against atherosclerosis

**Figure 8:**
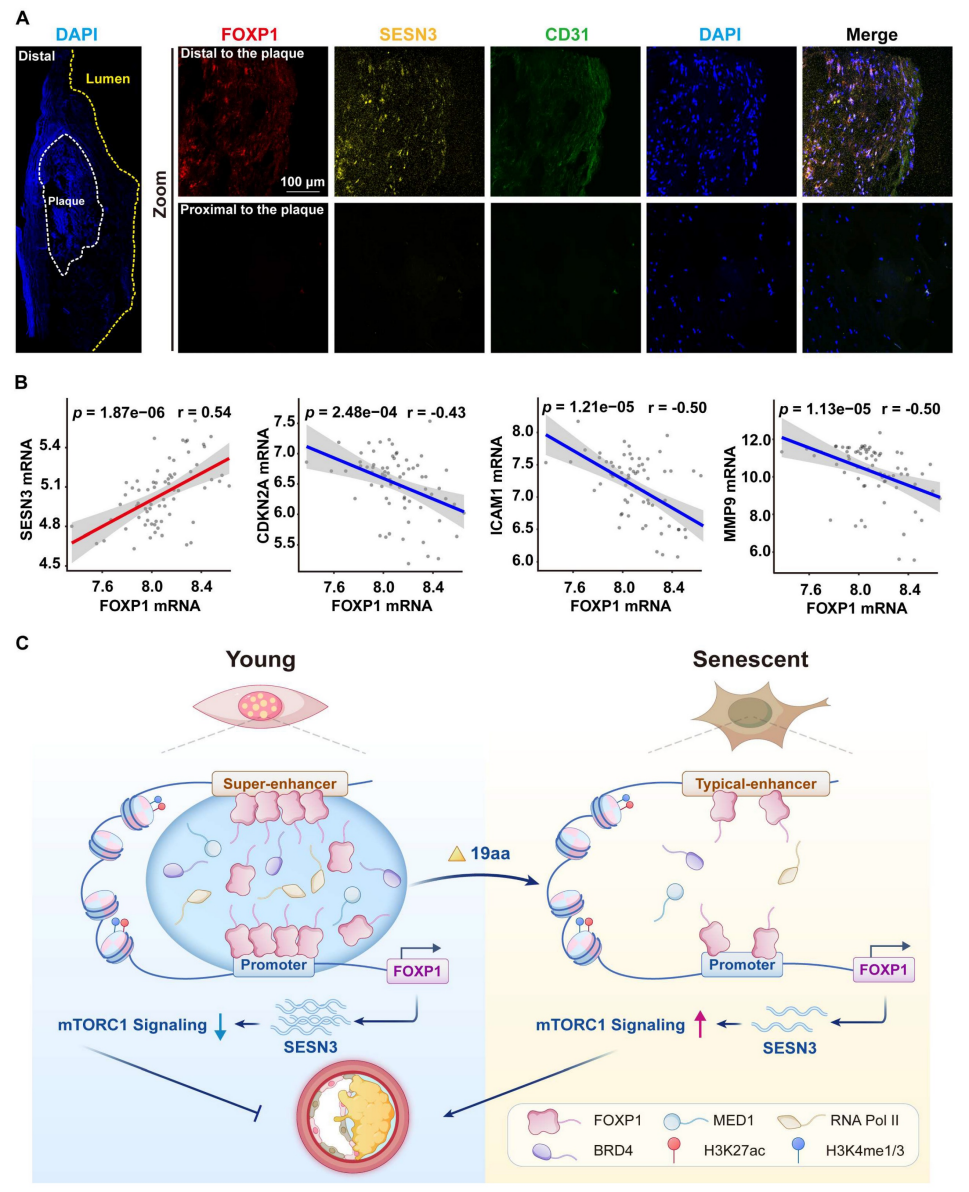
**The expression of FOXP1 was positively correlated with SESN3 in human carotid plaques. (A)** FISH images of human carotid atherosclerotic plaques indicate that the expression of FOXP1 and SESN3 occurs in both distal and proximal regions (*n* = 3). **(B)** Correlation analysis of FOXP1 with SESN3, inflammatory markers, and senescent markers in plaque tissue of GSE100927 (*n* = 69) 75, statistical analysis was performed by the Spearman correlation. **(C)** The schematic diagram illustrates that FOXP1 undergoes phase separation at its super-enhancer, recruiting transcription coactivators to form condensates. These condensates, in turn, facilitate binding with the SESN3 promoter and inhibit the mTORC1 signaling pathway, thereby delaying endothelial cell senescence.

In this study, RNA FISH demonstrated that the expression of FOXP1 and SESN3 was upregulated in the endothelium of distal regions of human carotid atherosclerotic plaques compared to proximal regions (Figure 8A). Moreover, the expression of FOXP1 was positively correlated with SESN3 and negatively correlated with CDKN2A, MMP9, and ICAM1 in human atherosclerosis plaques (Figure 8B). These results suggest that FOXP1 and SESN3 have a potential protective effect against atherosclerosis.

## Discussion

The information theory of aging proposes that the loss of epigenetic information is the primary driver of aging 45. Vascular endothelial cell senescence leads to endothelial dysfunction, which in turn accelerates the formation and development of atherosclerotic plaques 3. In this study, we utilized CUT&Tag, ATAC-seq, and RNA-seq technologies to comprehensively characterize the super-enhancer landscape, chromatin accessibility, and gene expression patterns during the process of vascular endothelial cell senescence. Our findings revealed that the senescent endothelial cells undergo significant alterations in their epigenome and chromatin accessibility. Specifically, the levels of H3K27ac in senescent cells were notably diminished, whereas chromatin accessibility was increased. Further integrated analysis of CUT&Tag and RNA-seq data revealed that FOXP1 functions as a super-enhancer-driven core transcription factor in endothelial cells, and is enriched in the gain enhancer of young endothelial cells, exhibiting upregulation in the intima of young mice. Moreover, our data support the notion that FOXP1 undergoes phase separation dependent on 357-375 aa, which delays endothelial cell senescence by driving the transcriptional activation of SESN3 and subsequently inhibiting the mTORC1 signaling pathway (Figure 8C). Thus, targeting transcriptional activation mediated by FOXP1 phase separation presents a promising strategy for intervening in endothelial cell senescence.

Super-enhancers frequently regulate cell identity genes and are often mutated, which is associated with complex traits and genetic diseases 46, 47. We previously reported that LINC01503, driven by a super-enhancer, promotes the progression of esophageal squamous cell carcinoma 48. Although the significant role of super-enhancers in various diseases has been widely reported 47, 49, 50, their precise mechanism in regulating vascular endothelial cell senescence remains unclear. Recent studies have indicated that the transcriptional coactivators BRD4 and MED1 can form phase-separated droplets at super-enhancers 8. MED1-IDR droplets can compartmentalize and concentrate the transcription apparatus from nuclear extracts, thereby controlling the expression of key genes 8. We discovered that inhibiting the activity of FOXP1's super-enhancer results in the disruption of FOXP1 LLPS, suggesting that phase separation of FOXP1 occurs at its super-enhancer. At the molecular level, the key amino acids responsible for the phase separation of FOXP1 are crucial for maintaining its function in delaying endothelial cell senescence. Mechanistically, FOXP1 binds to the promoter region of the target gene SESN3, activating its expression and subsequently suppressing the mTORC1 signaling pathway, a pivotal process in endothelial cell senescence. Our results provide the first evidence of a connection between super-enhancer and endothelial cell senescence.

Due to the collision of various proteins and nucleic acid molecules, the cell-like structure is ultimately formed through multivalent weak interactions, resulting in the compartmentalization within the cell 37, 51. An increasing number of studies suggest a close association between abnormal phase separation and the onset and progression of various diseases. Specifically, the phase separation of FUS has been identified as a key biological process in the senescence of hematopoietic stem cells. Targeting this abnormal phase separation may represent a significant approach to delaying hematologic aging and addressing aging-related pathologies 52. A recent study has reported that the transcriptional activator SGF29 recruits transcriptional regulatory elements to form biological condensates through phase separation, thereby accelerating cell senescence 53. In this study, we identified that the 19 phase-forming amino acids essential for FOXP1 LLPS were located at IDR1, which maintains the ability of FOXP1 to delay endothelial cell senescence. Moreover, by integrating epigenetic regulation with phase separation theory, our study not only expands the scope of vascular aging research through a multidisciplinary framework, but also unveils novel avenues to explore "epigenetic-phase separation" mechanisms in vascular aging-related diseases.

Forkhead box proteins P (Foxps) are large modular transcription factors that bind to DNA via their highly conserved forkhead DNA-binding domain 54. FOXP1, known for controlling cell differentiation, proliferation, and development 13, 55-57, has been implicated in protecting against pathological cardiac remodeling and improving cardiac dysfunction 58. Previous studies have reported that transcriptional activity of FOXP1 is modulated by tissue-specific homo- and heterodimeriszation via a leucine zipper motif 54. We identified that the phase separation of FOXP1 depends on its 357-375 aa, which are located within the leucine zipper motif. This suggests that the dimerization of FOXP1 is potentially necessary for and may even facilitate its condensation. A previous study demonstrated that the DNA-binding domain of basic leucine zipper (bZIP) prevents FUS-CREB3L2 liquid condensates from transiting into a gel state, ensuring the highly efficient activation of low-grade fibromyxoid sarcoma-specific genes 59. FOXP1 proteins differ in that they are capable of forming homodimers or heterodimers with subfamily members. The dimerization domain is localized to an evolutionarily conserved leucine zipper motif. Therefore, the defective anti-senescence function of FOXP1 (∆357-375) may result from both the abolition of phase separation and the impact on dimerization. Here, our study offers a novel finding that FOXP1-mediated phase separation sustains the ability to delay endothelial cell senescence, thereby contributing to endothelial homeostasis and anti-atherosclerosis. However, further investigation is required to understand the impact of FOXP1 LLPS disruption on endothelial cell senescence and atherosclerosis *in vivo*, as well as the peptide targeting FOXP1 LLPS.

SESN3 functions as a critical antioxidant stress protein that negatively regulates the mTORC1 signaling pathway 44, 60. mTORC1 activity stimulates protein and lipid biosynthesis by phosphorylating p70 Ribosomal protein S6 kinase (p70S6K) and the eukaryotic translation initiation factor 4E-binding proteins (4E-BPs) 61. Previous studies have demonstrated that mTORC1 activity increases with age, inhibiting mTORC1 can extend both lifespan and healthspan in yeast, worms, and mice 62-64. In this study, SESN3 exhibited high expression in young endothelial cells, and its downregulation markedly promoted endothelial cell senescence. FOXP1 phase separation inhibited the downstream effectors of mTORC1 (p70S6K and S6). Overall, our findings provided evidence demonstrating the activation of SESN3 expression and the inhibition of mTORC1 signaling through FOXP1 phase separation.

In conclusion, we characterized the genomic landscape of endothelial cell senescence and revealed the critical role of the super-enhancer-driven core transcription factor FOXP1 in delaying endothelial cell senescence, alleviating endothelial dysfunction and reducing atherosclerosis. Notably, FOXP1 forms LLPS condensates in young endothelial cells, which sustain its ability to resist endothelial cell senescence. We further found that FOXP1 promotes the expression of its downstream target gene SESN3 through phase separation, thereby suppressing the mTORC1 signaling pathway. These findings not only clarify the essential role of FOXP1 in maintaining endothelial homeostasis but also provide a direct target for the development of small-molecule drugs based on transcription factor features or phase separation. Notably, our work highlights the potential of SESN3-targeted agents as novel intervention method for atherosclerosis, thereby offering a promising therapeutic strategy to inhibit atherosclerotic progression by delaying endothelial cell senescence.

## Methods

### Patients and tissue specimens

Human carotid atherosclerotic plaques were obtained from patients undergoing carotid endarterectomy surgery at Affiliated Huizhou First Hospital, Guangdong Medical University. All patients or their guardians have signed an informed consent form prior to participation in the study. This study was carried out in accordance with the principles of the Declaration of Helsinki, and the research protocol was approved by the Ethics Committee of the Affiliated Huizhou First Hospital, Guangdong Medical University under ethical approval number KYLL-2024-126-01.

### Mouse experiments

All animal procedures were approved by the Institutional Animal Care and Use Committee of Guangdong Medical University under approval number GDY2004009. C57BL/6J mice and ApoE KO mice were purchased from Shanghai Model Organisms Center, Inc. For AAV9 generation and injection, AAV9 vector was constructed by WZ Biosciences Inc. ApoE KO mice were restrained, and their tails were injected with AAV9-con or AAV9-FOXP1 at a dose of 5 × 10^11^ viral particles in a 100 μL volume of sterile PBS. Subsequently, they were fed a 12-week Western Diet (Guangdong medical laboratory animal center, WD) before being sacrificed. For studies on endothelial cell senescence and atherosclerosis, mice were anesthetized using continuous inhalation of 2.5% isoflurane gas. Hearts were then isolated from the mice and processed for frozen embedding. Aortas were collected from the base of the ascending aorta to the iliac bifurcation for the measurement of aortic *en face* senescence and atherosclerosis. The aortic roots and whole aortas were stained with SA-β-gal and Oil Red O to quantify the degree of senescence and atherosclerosis. The quantification was performed using ImageJ. For the isolation of endothelial RNA, mice were anesthetized using continuous inhalation of 2.5% isoflurane gas. The thoracic cavity was then opened to expose the heart and aorta. The aorta was longitudinally incised, and endothelial cells were lysed *in situ* by perfusing the luminal surface with TRIzol delivered from a syringe for 10 seconds. The TRIzol effluent was immediately collected and processed for total RNA extraction following the manufacturer's instructions.

### Antibodies and reagents

The following antibodies and reagents were used: anti-H3K27ac (Abcam, ab4729, 1:50 for CUT&Tag, 1:1000 for IF), anti-H3K4me1 (Abcam, ab8895, 1:50 for CUT&Tag, 1:1000 for IF), anti-H3K4me3 (Abcam, ab8580, 1:50 for CUT&Tag, 1:1000 for IF), anti-H3K9me3 (Abcam, ab8898, 1:500), anti-FOXP1 (Abclonal, A23442, 1:200 for IF, 1:1000 for WB), anti-SESN3 (Proteintech, 11431-2-AP, 1:200 for IF, 1:1000 for WB), anti-His (Thermo, MA1-21315, 1:1000), anti-CD31 for mice (BioLegend, 102502, 1:150), anti-GAPDH (Proteintech, 60004-1, 1:5000), Goat anti-Rabbit IgG (Vazyme, Ab207, 1:50 for CUT&Tag), Goat anti-Mouse IgG, HRP (Beyotime, A0216, 1:5000), Goat anti-Rabbit IgG, HRP (Beyotime, A0208, 1:5000), anti-mTOR (CST, 2983T), anti-p-mTOR (MCE, HY-P80837), anti-S6 (HuaBio, HA601214, 1:1000), anti-p-S6 (HuaBio, HA721589, 1:1000), anti-P70-S6K (HuaBio, HA722520, 1:1000), anti-p-P70-S6K (HuaBio, HA721803, 1:1000), anti-IgG (Proteintech, 30000-0-AP, 1:5000), anti-ICAM1 (Abcam, ab119871, 1:200), anti-VCAM1 (CST, 39036S, 1:400), anti-p16 (CST, D7C1M, 1:50 for IF, 1:1000 for WB), anti-DYKDDDDK (Thermo, MA1-91878, 1:400), anti-MED1 (Abclonal, A1724, 1:400), anti-RNA pol II (HuaBio, HA721880, 1:400), anti-CTCF (HuaBio, ET1703-90, 1:400), Goat anti-Mouse Alexa Fluor Plus 488 (Proteintech, RGAM002, 1:1000), Goat anti-Rabbit Alexa Fluor Plus 555 (Proteintech, RGAR003, 1:1000), Goat anti-Rat Alexa Fluor Plus 647 (Abbkine, A23640, 1:1000), Goat anti-Rabbit Alexa Fluor Plus 488 (Proteintech, RGAR002, 1:1000), Goat anti-Mouse Alexa Fluor Plus 647 (Proteintech, SA00014-10, 1:1000), Goat anti-Mouse Alexa Fluor Plus 555 (Proteintech, RGAM003, 1:1000), Fluoromount-G (southernbiotech, 0100-01), His-tag Protein Purification Kit (Beyotime, P2226), Phanta mix (Vazyme, P525-02-AA), SYBR qPCR mix (Vazyme, Q712), Golden Gate mix (Abclonal, RM20590), Seamless mix (Abclonal, RM20523), BsmBI (NEB, R07395), KpnI (NEB, 1618), XhoI (NEB, 1635), MluI (NEB, 1619), NheI (NEB, 1622), EcoRI (NEB, 1611), Stu I (Yeasen, 15031ES), Lambda Exonuclease (Beyotime, D7084), goat serum (Invitrogen, 16210064), SDS-PAGE Gel Kit (Bio-Rad, 1610185), Enhanced BCA Protein Assay Kit (Beyotime, P0010), BeyoClick EdU Kit (Beyotime, C0078S), CELLSAVING (NCM Biotech, C40100), DMEM (BaiDi Biotechnology, L100-500), TRIzol (Invitrogen, 15596018CN), FITC-labeled Dextran (Beyotime, ST2930), RIPA (Beyotime, P0013B), the Hyperactive Universal CUT&-Tag Assay Kit for Illumina (Vazyme, TD904), VAHTS DNA Clean Beads (Vazyme Biotech, N411), Lipo8000 (Beyotime, C0533FT), polyethyleneimine (PEI) (Servicebio, G1802), Lipofectamine™ RNAiMAX (Invitrogen, 13778100), PMSF (Beyotime, ST507), endothelial cell growth medium (ScienCell, 1001), 0.25% Trypsin-EDTA (Gibco, 25200056), Oil Red O (Sigma, 1320-06-5), Senescence β-Galactosidase Staining Kit (Beyotime, C0602), Double-Luciferase Reporter Assay Kit (Beyotime, RG029S), TruePrep DNA Library Prep Kit V2 (Vazyme, TD501), JQ-1 (MCE, HY7869), THZ1 (MCE, HY80013), SplintR buffer (NEB, B0375S), SplintR Ligase (NEB, M0375L), glycerol (Sigma-Aldrich, G5516), BSA (Sigma-Aldrich, B2064), EquiPhi29 buffer (Thermo Fisher Scientific, A39391), Equi Phi29 DNA polymerase (Thermo Fisher Scientific, A39391), dNTP (Thermo Fisher Scientific, R0182) and 1,6-hexanediol (Sigma, 240117).

### Cell culture

Primary HUVECs were purchased from ScienCell, and cultured in endothelial cell growth medium supplemented with 5% FBS, 1% ECGS, 1% penicillin/streptomycin at 37 ℃ under a humidified atmosphere containing 5% CO_2_. A replicative senescence model was established by passaging the HUVECs when they reached 80%-90% density, typically every 2 or 3 days.

### CUT&Tag-seq and CUT&Tag qPCR

The library preparation for CUT&Tag was performed as previously reported 65. We collected 1.5 × 10⁵ HUVECs at different stages using 0.05% trypsin digestion and performed subsequent library construction with the Hyperactive Universal CUT&Tag Assay Kit. Briefly, cells were washed with 500 μL of wash buffer and centrifuged at 600 × g for 5 min at room temperature. The cell pellet was resuspended in 100 μL of wash buffer. A total of 10 μL of ConA beads was washed twice with 100 μL of binding buffer, and then added to the cells, followed by incubation at room temperature for 10 min. After removal of the supernatant, the cells bound to the magnetic beads were resuspended in 50 μL of antibody buffer containing 1 μg of antibodies (H3K27ac, H3K4me1, H3K4me3) and incubated overnight at 4 ℃. Subsequently, 1 μL of goat anti-rabbit IgG diluted in 50 μL of Dig-wash buffer was added to the cells and incubated with rotation for 1 h at room temperature. The cells were gently washed three times with 200 μL of Dig-wash buffer, and then 2 μL of pA/G-Tnp and 98 μL of Dig-300 buffer were added to each sample. After incubating at room temperature for 1 h, the samples were gently washed three times with 200 μL of Dig-300 buffer. Next, 10 μL of 5 × Tagmentation DNA Buffer (TTBL) mixed with 40 μL of Dig-300 buffer was added to each sample, and the samples were incubated at 37 ℃ for 1 h. DNA extraction was performed by adding 5 μL of proteinase K, 100 μL of buffer L/B, and 20 μL of DNA extraction beads, followed by incubation at 55 ℃ for 10 min. For library amplification, 15 μL of extracted DNA was mixed with 25 μL of 2 × CAM, P5 Primer, and P7 Primer. The H3K27ac library was amplified for 13 cycles, while the H3K4me1 and H3K4me3 libraries were amplified for 11 cycles. To purify the PCR products, 2 × VAHTS DNA Clean Beads was added, followed by incubation at room temperature for 5 min. After purification, paired-end sequencing was performed on the NovaSeq 6000 platform with three biological replicates at Novogene Biotech Co., Ltd.

For CUT&Tag qPCR, added 5 μL Stop Buffer to the resuspended DNA Extract Beads Pro from the DNA extraction step, mix thoroughly, and incubate at 95 ℃ for 5 min. Centrifuge instantaneously, place the 8-plex tubes on a magnetic rack, and transfer the supernatant to a new 8-plex tube after the solution is clarified (30 s - 2 min). The supernatant obtained was the fragmented DNA, which was used for qPCR. The primers used for CUT&Tag-qPCR are listed in Table S3.

### Data analysis of CUT&Tag-seq

For CUT&Tag-seq data, reads were mapped to the human reference genome (hg19) using Bowtie2 (version 2.4.5) 66. The aligned BAM files were subjected to quality control using SAMtools 67, and only uniquely mapped reads were retained for further analysis. Significant peaks were called using MACS2, with the exception of the -q 0.01 parameter, all other parameters were set to default values 68. BigWig files were generated from the BAM files using bamCoverage from deepTools 69, with the following parameters: -binSize 10 -effective GenomeSize 2864785220 and normalized using RPKM. CUT&Tag signal of H3K27ac was used to define super-enhancers, ROSE was employed for enhancer and super-enhancer identification, along with the annotation of relevant genes 7. Spearman correlation coefficient analysis based on the read coverages for genomic regions for Bigwig files was performed using deepTools multiBigwigSummary 69. Peak distributions were analyzed by ChIPseeker package 70.

### ATAC-seq

5 × 10^4^ HUVECs at different stages were collected by 0.05% trypsin digestion and resuspended the samples in 1 × phosphate-buffered saline (PBS). The cells were centrifuged at 500 g, 4 ℃ for 5 min, and the cell pellet was then resuspended in 50 μL of ice-cold lysis buffer containing 10 mM Tris-HCl (pH 7.4), 10 mM NaCl, 3 mM MgCl_2_, and 0.1% (v/v) Igepal CA-630. The cell suspension was placed on ice for 2 min to allow cell lysis, followed by centrifugation at 500 × g for 5 min at 4 °C to collect the nuclei. The lysed cells were then transferred to sterile PCR tubes and subjected to transposition reaction and library construction using the TruePrep DNA Library Prep Kit V2. For the transposition reaction and library construction, the lysed cell samples were mixed with 5 × TTBL, TTE-Mix V50, and ddH_2_O according to the kit instructions. The mixture was heated at 37 °C for 30 min to facilitate the fragmentation reaction. Afterward, 5 × TAB, PPM, P5 Primer, and P7 Primer were added to the enzymatically treated products purified using VAHTS DNA Clean Beads. The DNA library was obtained by performing 15 cycles of PCR amplification. Subsequently, the library was purified and subjected to paired-end sequencing on the NovaSeq 6000 platform with two biological replicates at Novogene Biotech Co., Ltd.

### Data analysis of ATAC-seq

For ATAC-seq data, reads were aligned to the human reference genome (hg19) using Bowtie2 (version 2.4.5) 66. The aligned BAM files underwent quality control using SAMtools 67, and only uniquely mapped reads were retained. PCR duplicates were removed using Picard (http://broadinstitute.github.io/picard/) for subsequent analysis. Significant peaks were called using MACS2, with the exception of the -q 0.01 parameter, all other parameters were set to default values 68. BigWig files were generated from the BAM files using bamCoverage from deepTools 69, with the following parameters: -binSize 10 -effectiveGenomeSize 2864785220 and normalized using RPKM. Peak distributions were analyzed by ChIPseeker package 70.

ATAC-CUT&Tag peak overlap: Overlap analysis of ATAC-seq and H3K27ac CUT&Tag-seq peaks was performed using the bedtools intersect -a ATAC_peaks.bed -b H3K27ac_peaks.bed -c > overlap_counts.bed 71. The command was used to identify the regions of overlap between the two peak sets, and the output was saved in a BED format file. The percentage of ATAC-seq peaks that overlapped with H3K27ac CUT&Tag-seq peaks was calculated as the total length of overlapping regions divided by the total length of ATAC-seq peaks.

### ATAC-Seq fragment plot

The insert size distribution plot for ATAC-seq was generated by extracting fragment length information from the ninth column of the BAM file using the Samtools view command 67. The extracted fragment length information was then imported into R statistical software for visualization. The insert size distribution plot was generated by plotting the frequency of each fragment length on the y-axis against the fragment length on the x-axis using the ggplot2 package in R statistical software.

### RNA-seq

TRIzol reagent was utilized for the isolation of total RNA according to the manufacturer's instructions. Subsequently, the extracted RNA was sent to Guangzhou Epibiotek Co., Ltd. for further processing, including cDNA library preparation and Illumina sequencing using a NovaSeq 6000 platform with paired-end read configuration.

### Data analysis of RNA-seq

For RNA-Seq data, reads were mapped to the human reference genome (hg19) using hisat2 (version 2.1.0). SAMtools was employed to perform quality control on the aligned BAM files, retaining only uniquely mapped reads 67. The quantification of reads was conducted using featureCounts. TPM values that represent gene expression were generated using R statistical software. DESeq2 was utilized for differential expressed gene analysis with Log2 (fold change) ≥ 1 and *P* value < 0.05. BigWig files were generated from the BAM files using bamCoverage from deepTools 69.

### Principal component analysis

The plotPCA tool of deepTools was utilized to analyze the enhancer, promoter, and super-enhancer profiles of HUVECs at different stages.

### Identification of differential enhancer, promoter, super-enhancer

For different stages of HUVECs, the bedtools subtract command was used to identify specific-enhancer, promoter, or super-enhancer. This command was employed to identify differential regions between two sets of peaks. For instance, regions in set A were subtracted from regions in set B, the parameter -A was used to remove all overlapping regions. Taking super-enhancers as an example for finding gain or loss elements: firstly, all BAM files were merged, and the MACS2 call peak algorithm was applied to identify the peaks of super-enhancer. The count value of super-enhancer peaks in the BAM files was then calculated. Subsequently, the differential expression analysis was performed using the DESeq2 package in R statistical software. Differential peaks were determined based on the criteria of |Log2 fold change| > 1 and *P* value < 0.05. Upregulation indicated gain, while downregulation indicated loss. Finally, the HOMER software was employed to annotate the genes associated with the differential peaks.

### Gene ontology (GO) analysis

The R package clusterProfiler was utilized to identify significantly enriched GO biological processes, applying a *p*-value cutoff of 0.05 and a *q*-value cutoff of 0.05.

### CRC identification

Coltron, a Python package, was used to build transcriptional regulatory networks by integrating H3K27ac CUT&Tag data as the previous method (https://pypi.python.org/pypi/coltron) 72. In brief, the interaction network of SE-associated TFs was constructed using in-degree and out-degrees to measure node connectivity. This quantifies the degree of in-degree and out-degree regulation of core transcription factors regulated by super-enhancers, with in degree representing the ability to which transcription factors are regulated by other core transcription factors and out degree representing the ability of transcription factors to regulate other core transcription factors.

### Plasmid construction, lentiviral packaging and RNA interference

FOXP1 cDNA was derived from a HUVEC cDNA library and then homologously recombined into pcDNA3.1-EGFP, pET-28a (+), and pLVX vectors using the Seamless Cloning Kit for the purposes of transient transfection, protein purification, and lentiviral packaging, respectively. Subsequently, critical fragments were deleted through reverse PCR. Lentivirus was packaged in HEK293T cells. Briefly, lentiviral vector, packaging plasmid (psPAX2) and coat protein plasmid (pMD2.G) were transfected at 3:2:1 ratio by polyethyleneimine (PEI) reagent. Viral particles were collected 48 h after transfection, 0.45µm filtered, and concentrated with 100K Centrifugal Filter Unit. For RNA interference, Lipofectamine™ RNAiMAX was used according to the manufacturer's instructions. SiRNA and RNAiMAX were mixed using Opti-MEM, and the mixture was incubated at room temperature for 15 min and then dropped evenly into HUVECs with approximately 70% confluency. Three siRNAs targeting human FOXP1 and a nontargeting control siRNA were purchased from RiboBio (Guangdong, China), and two siRNAs targeting human SESN3 were purchased from Tsingke Biotech (Beijing, China). The specific siRNA sequences can be found in Table S5.

### dCas9-KRAB CRISPRi and sgRNA construct design

To modulate enhancer activities, we designed sgRNAs and subsequently integrated them into dCas9-KRAB lentiviral vectors to facilitate the repression of enhancers. The sgRNA sequence is listed in Table S4. To suppress endogenous FOXP1 expression, we devised a multiplexed CRISPRi strategy. Three sgRNAs were designed targeting the promoter region of FOXP1 and were ligated to the dCas9-KRAB vector using tRNA sequences in tandem. These vectors were then packaged into lentivirus and used to infect HUVECs. The sgRNA sequences can be found in Table S4.

### Padlock probe (PLP) hybridization and ligation

Cells were seeded on slides, fixed with 4% PFA for 30 min, and dehydrated with 70%, 80%, and 100% ethanol for 5 min each. ImmEdge hydrophobic barrier pen was used to create a reaction area. After digesting with 0.5 U/μL blunt-end restriction enzyme StuⅠ at 37 ℃ for 1 h, washed three times with 1 × DEPC-PBST. To digest the DNA double strands into single strand, 0.2 U/μL Lambda Exonuclease digestion was performed at 37 ℃ for 30 min, followed by three washes with 1 × DEPC-PBST. To hybridize the PLPs with target single-stranded DNA, 0.1 μM of phosphorylated PLPs in 6 × SSC and 10% formamide was added on the slides and incubated for 4 h at 37 °C. After washing three times with 1 × DEPC-PBST, PLPs was ligated by applying a ligation mix containing 1 × SplintR buffer, 2.5 U/μL SplintR Ligase, 50% glycerol, and 0.2 μg/μL BSA, and incubated for 1 h at 37 °C. Then the slide was washed three times with 1 × DEPC-PBST. The probe sequences can be found in Table S5.

### Rolling circle amplification (RCA)

First, 0.1 μM of RCA primers in 6 × SSC and 10% formamide was added to the reaction area and incubated for 1 h at 37 °C, allowing hybridization with PLPs. RCA was initiated by applying a reaction mix in DEPC-H_2_O containing 1 × EquiPhi29 buffer, 0.1 U/μL Equi Phi29 DNA polymerase, 1 mM dNTP, 5% glycerol, 0.2 μg/μL BSA, and 1 mM DTT to the reaction area and incubated overnight at 37 °C, then washed three times with 1 × DEPC-PBST. Finally, RCA products (RCPs) were visualized by being hybridized with 0.1 μM fluorophore-conjugated detection probes in 6 × SSC and 10% formamide for 30 min at room temperature. The slides were ready for imaging after being mounted with SlowFade Gold Antifade Mountant containing 0.5 µg/mL DAPI.

### SA-β-gal staining

SA-β-gal staining was performed using the Senescence β-Galactosidase Staining Kit following the manufacturer's instructions. Briefly, cells or tissue were PBS-washed and fixed in SA-β-gal fixing solution for 15 min at room temperature. After three washes with PBS, cells or tissue were incubated with the working solution at 37 ℃ overnight. Images were acquired at 100 × magnification using an Eclipse TS100 Inverted Microscope (Nikon Corporation) and analyzed using the ImageJ software (NIH) to quantify the percentage of cells undergoing senescence based on SA-β-gal signals.

### *In vitro* permeability assay

The FITC-Dextran transendothelial flux was quantitatively measured as an index of endothelial permeability. A density of 10^5^ cells/mL HUVECs was seeded on transwell membranes and grown until they formed a functional monolayer. Tracer FITC-dextran (1 mg/mL) was added to the upper chambers of the transwell system. The transfer of FITC-dextran across the HUVECs monolayers was quantified after incubation at 37 ℃ for 30 minutes, and samples were then collected from the lower chambers. The fluorescence was measured with a plate reader using 492 nm and 520 nm as the excitation and emission wavelengths, respectively.

### EdU assay

EdU staining was performed using the BeyoClick EdU Kit following the manufacturer's instructions. Briefly, cells were fixed and permeabilized, incubated with the addition of the thymidine deoxyriboside analogue EdU, and labelled with Alexa Fluor 488 or 594 by a subsequent click reaction. The resulting proliferating cells exhibited intense green or red fluorescence under fluorescence microscopy. Images were acquired at 100 × magnification using an EVOS Live-Cell Imager System (Thermo Fisher) and analyzed using the ImageJ software to quantify the percentage of proliferating cells.

### Tube formation

For tube formation, 70 μL matrigel was evenly applied to the bottom of the 96-well plate and after curing at 37 °C for 30 min, 8 × 10^3^ cells were seeded onto the matrigel and cultured. The endothelial cells were observed to form tubes in about 4-6 h, and images were collected and quantitatively analyzed by ImageJ software.

### Immunofluorescence

Cells or frozen tissue sections were fixed with 4% paraformaldehyde at room temperature for 15 min, followed by three washes with PBS. Samples were then blocked with 5% goat serum and 0.3% Triton X-100 in PBS at room temperature for 1 h. Antibodies were diluted in 0.3% Triton X-100 in PBS and incubated overnight at 4 °C. After three washes with PBS, samples were incubated with secondary antibodies and DAPI for 1 h at room temperature, washed three additional times with PBS. Samples were mounted with Fluoromount-G. Images were captured using a confocal microscope (Leica SP8). Detailed information of antibodies was listed in antibodies and reagents.

### Western blot

Tissues or cells were lysed in a lysis buffer suitable for Western blot, which included a protease inhibitor PMSF at a 1:100 dilution. The lysates were then centrifuged at 10,000 rpm for 10 min at 4 °C, and the supernatants were collected for analysis. Protein concentrations were measured using a BCA protein assay. The total proteins were separated using 12.5% SDS-PAGE and subsequently transferred onto PVDF membranes. Signals were detected using a chemiluminescent HRP substrate.

### Quantitative RT-PCR

The TRIzol reagent was used to isolate total RNA following the manufacturer's instructions. A total of 1 μg of RNA was reverse-transcribed into complementary DNA (cDNA) using the PrimeScriptTM RT Master Mix. Quantitative PCR was conducted using the qPCR SYBR Master Mix on a QuantStudio system, with GAPDH serving as the internal control. Quantification was performed using the comparative ∆∆CT method. The primer sequences used were listed in Table S3.

### Luciferase reporter assay

The enhancer elements were amplified by PCR from the genomic DNA extracted from HUVECs and cloned into the pGL3-Promoter luciferase reporter vectors. The primers are included in Table S3. The luciferase reporter plasmid and an internal control plasmid Renilla luciferase vector was transfected into HEK293T cells using Lipo8000. After 48 h of transfection, the luciferase activity was measured by the Double-Luciferase Reporter Assay Kit.

### Live-cell imaging

HUVECs-SV40 or HEK293T cells were seeded in glass bottom dishes. Transfection of pcDNA3.1-EGFP-FOXP1 and mutant plasmids into the cells was performed using Lipo8000, and imaging was conducted 36 h later using confocal microscopy (Leica SP8) at channel 488 nm.

### 1,6-hexanediol treatment

10% 1,6-hexanediol was diluted in PBS and added to the cells or recombinant protein solution for 5 min, then cells were fixed with 4% PFA for Immunofluorescence staining or live-cell imaging directly.

### FRAP

FRAP assays were performed using a Leica SP8 confocal microscope. For FRAP experiments *in vivo*, after transfection of EGFP-FOXP1 and mutant plasmids in HUVECs-SV40 or HEK293T cells for 24 h, turn on the FRAP mode of the microscope, a single control image was acquired before bleaching, select the bleached area to set the fluorescence to 100% laser power (488 nm laser), bleaching was continued for 5 s in the middle and then withdraw the fluorescence and the recovery period was set to 60 s for observing the changes in the droplets and recording fluorescent signal. For FRAP experiments *in vitro*, droplets were formed with 26 μM EGFP-FOXP1-IDR1 recombinant protein in the presence of 125 mM NaCl and 7.5% PEG 4000, the FRAP steps are as previously described.

### Protein purification

The *E. coli* prokaryotic system was used to express recombinant proteins, EGFP-FOXP1 and EGFP-FOXP1-IDR1 sequences were ligated into pET-28a (+)-6 × His vectors to transform BL21 (DE3) competent cells. After coating the plate, single colonies were picked and supplemented with 0.3 mM isopropyl-β-d-thiogalactoside and incubated overnight at 16 ℃. The bacterial pellets were collected, and then sonicated in a non-denaturing lysis buffer with protease inhibitors. The lysates were spun and added to BeyoGold His-tag Purification Resin which had been pre-equilibrated with 5 volumes of the same buffer. The slurry was poured into a column, washed with buffer containing 10 mM imidazole and eluted with elution buffer containing 20 mM imidazole. The flow-through solution was collected, and analyzed by SDS-PAGE gel to check the protein expression. The protein solution was transferred into the dialysis bag, and dialyzed at 4 ℃ for 12 h in 1 L dialysis buffer (50 mM Tris-HCl, pH = 7.5, 125 mM NaCl, 10% glycerol and 1 mM DTT) with rotation. The dialyzed recombinant protein was concentrated using ultrafiltration and then quantified by BCA.

### Optodroplet assay

The OptoDroplet assay was performed as described previously 73. Briefly, the fusion expression of the Cry2PHR structural domain, following FOXP1-IDR was transfected into HEK293T cells, which were then subjected to time-series scanning at a confocal microscope with a 488-nm laser. One image was captured every 20 s for a period of 10 min.

### dCas9-SunTag to visualize SESN3 promoter

The plasmid involved in the dCas9-SunTag system was a gift from Kang Tiebang Lab. The assay was performed as described previously 74. Briefly, 10 sgRNAs (sgARRAY) around the SESN3 promoter were cloned into a pGL3-sgRNA backbone via Golden Gate reaction. pTETON-dCas9-24*GCN4, pTETon-scFv-GCN4-sfGFP and sgARRAY were cotransfected into HEK293T cells for 6 h, and 0.5 μg/mL doxycycline was added into the medium. After 18 h, the cells were fixed for immunofluorescence staining. Primary antibodies were anti-FOXP1, secondary antibodies were Goat anti-Rabbit Alexa Fluor Plus 555. Images were captured using a confocal microscope (Leica SP8). The sequences of the sgARRAY are provided in Table S4.

### Statistical analysis

Statistical analysis was performed using GraphPad Prism 9.4.1. Data are presented as mean ± SD. Before conducting parametric testing, the normality of the distribution was confirmed using the Shapiro-Wilk test (*P* > 0.05), and the homogeneity of variance was verified using the Brown-Forsythe test (*P* > 0.05). Correlation analysis was conducted using Spearman coefficient. One-way ANOVA was used to test for the effect of multiple levels of a factor on quantitative data, and a two-tailed Student's *t*-test was used to compare the differences between the two groups. *P* < 0.05 was considered statistically significant.

## Supplementary Material

Supplementary figures.

Supplementary tables.

Supplementary movie 1.

Supplementary movie 2.

Supplementary movie 3.

## Abbreviations

BRD4: Bromodomain-Containing Protein 4
CUT&Tag: Cleavage Under Targets and Tagmentation
EdU: 5-Ethynyl-2'-deoxyuridine
FOXP1: Forkhead box protein P1
GO: Gene ontology
H3K27ac: Histone H3 lysine 27 acetylation
H3K4me1: Histone H3 lysine 4 monomethylation
H3K4me3: Histone H3 lysine 4 trimethylation
H3K9me3: Histone H3 lysine 9 trimethylation
HUVEC: Human umbilical vein endothelial cell
IDR: intrinsically disordered region
1,6-HD: 1,6-hexanediol
LLPS: liquid-liquid phase separation
mTORC1: Mechanistic target of rapamycin complex 1
SA-β-gal: Senescence associated β-galactosidase
SE: Super-enhancer
SESN3: Sestrin 3
siRNA: Small interfering RNA
TF: Transcription factor
