# Supplementary material for: Super-enhancer-driven core transcription factor FOXP1 delays endothelial cell senescence via phase separation-mediated SESN3 activation
Source: Theranostics. 2026 Jan 1;16(3):1386–409. doi: 10.7150/thno.119709 (PMC12679572; doi:10.7150/thno.119709)
Supplement: Supplementary file 1 — Supplementary figures. [file thnov16p1386s1.pdf]

Supplementary Material

Super-enhancer-driven core transcription factor FOXP1 delays endothelial cell senescence via phase separation-mediated SESN3 activation

Lushuang Mao[1,2], Zhao-fu Liao[1,2], Dong Tang[1,2], Yumin Qiu[3], Min Yang[4], Yanshang Li[4], Yituan Xie[5], Weimin Feng[6], Ze-jun Zheng[6], Xiao-meng Liu[1,2], Jing-ru Ye[1,2], Shui-hong Lu[1,2], Xin-bin Tang[1,2,6], Ming Shi[1], Yun-fei Qu[7], Heng Li[8], Zhu-guo Wu[7], Shun Xu[1,2], Xinguang Liu[1,2,6], Junjun Ding[9], Jian-jun Xie[6,✉], Jun Tao[3,✉], Xing-dong Xiong[1,2,6,7,✉]

1. Guangdong Provincial Key Laboratory of Medical Immunology and Molecular Diagnostics, The First Dongguan Affiliated Hospital, Guangdong Medical University, Dongguan 523808, P.R. China;
2. Dongguan Key Laboratory of Aging and Anti-Aging, Institute of Aging Research, Guangdong Medical University, Dongguan, P.R. China;
3. Department of Hypertension and Vascular Disease, The First Affiliated Hospital of Sun Yat-sen University, Guangzhou, P.R. China;
4. Department of Biochemistry and Molecular Biology, Shantou University Medical College, Shantou 515041, China;
5. Department of Neurosurgery, the Affiliated Huizhou First Hospital, Guangdong Medical University, Huizhou, Guangdong 516003, P.R. China;
6. Institute of Biochemistry & Molecular Biology, School of Basic Medical Sciences, Guangdong Medical University, Dongguan, P.R. China;
7. Cardiovascular Center, The First Dongguan Affiliated Hospital, Guangdong Medical University, Dongguan, P.R. China;
8. Department of Cardiovascularology, Dongguan Tungwah Hospital, Dongguan, 523808, P.R. China;
9. RNA Biomedical Institute, Sun Yat-Sen Memorial Hospital, Zhongshan School of Medicine, Sun Yat-Sen University, Guangzhou, P.R. China.

✉ Correspondence:

Xing-dong Xiong (xiongxingdong@gdmu.edu.cn)

Jun Tao (taojungz123@163.com)

Jian-jun Xie (xiejj0816@foxmail.com)

This word file includes:

Figure S1 to Figure S11

Movie 1 to Movie 3

Legends for table S1 to table S6

Other Supplementary Materials for this manuscript include the following:

Table S1 to S6

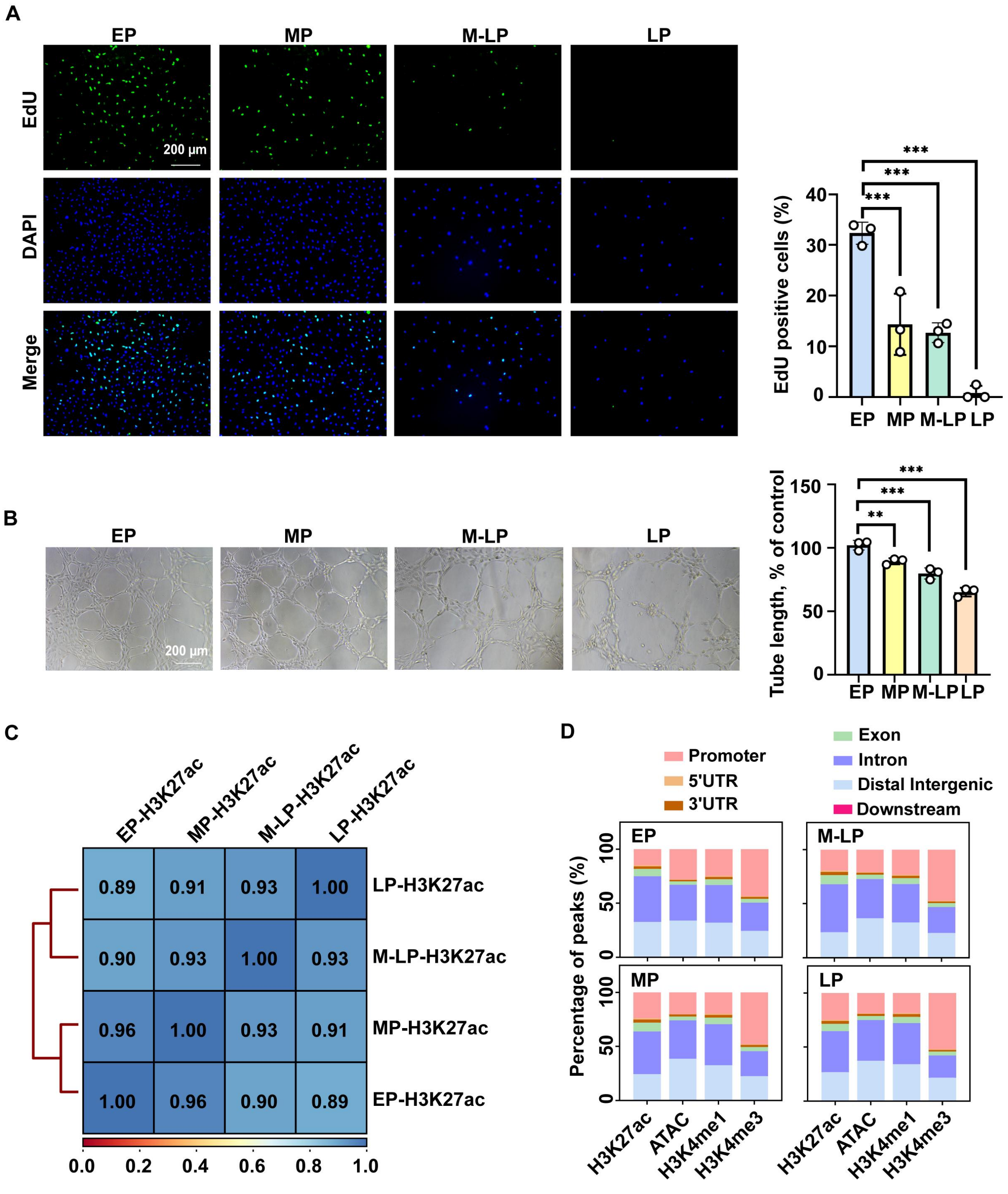

Figure S1. Successive generations of HUVECs undergo replicative senescence. (A) EdU assays were conducted to assess the proliferative capacity across four stages of HUVECs. **(B)** Tube formation assay was performed on four stages of HUVECs. **(C)** The correlation was analyzed using Deeptools for H3K27ac CUT&Tag across four stages of HUVECs. **(D)** Distribution patterns of H3K27ac, H3K4me1, H3K4me3, and ATAC peaks across the genome. Data are presented as means ± SD, one-way ANOVA, $^{**}P < 0.01$, $^{***}P < 0.001$.

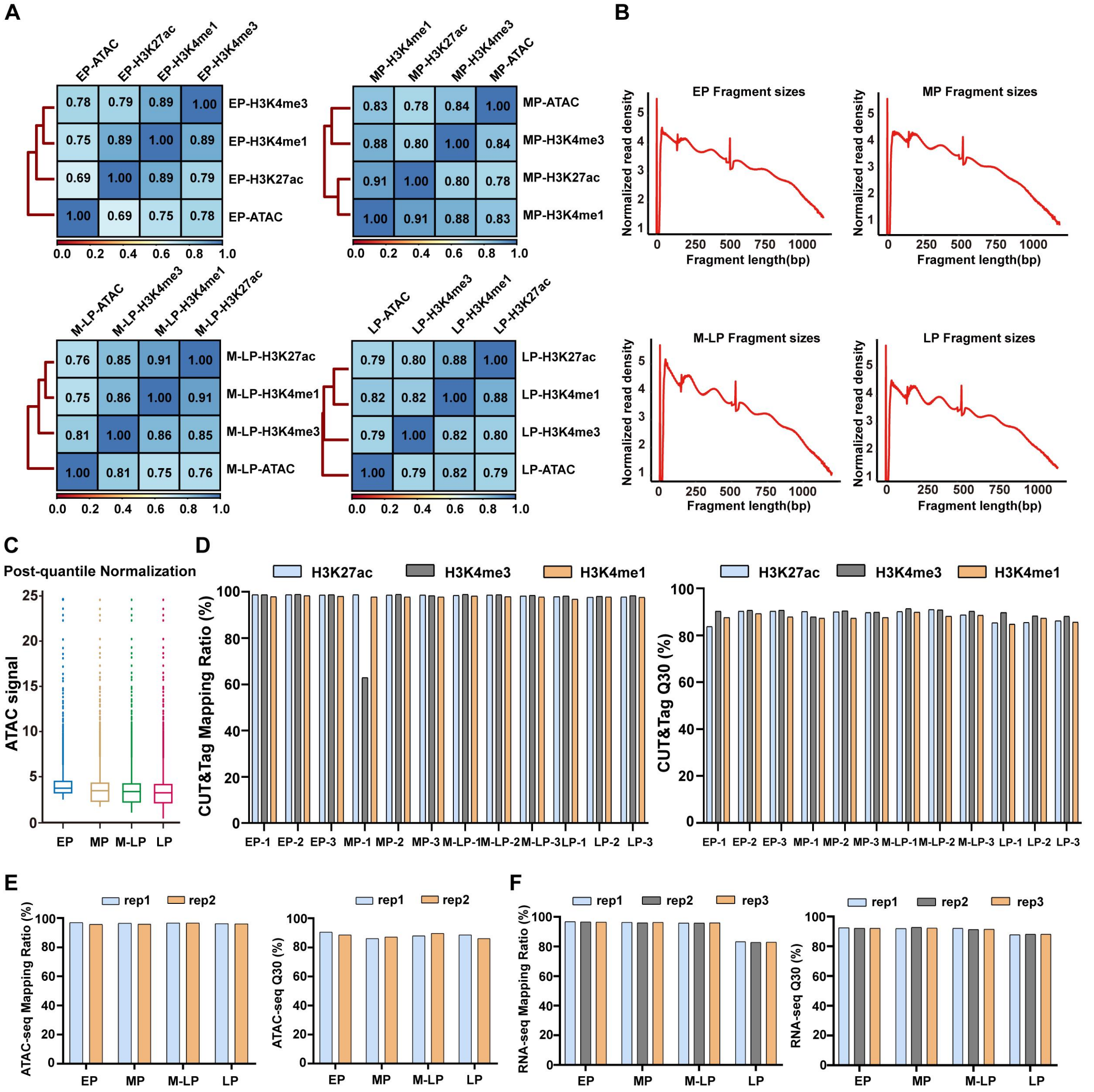

Figure S2. Quality analysis of sequencing data. (A) The correlation was analyzed using Deeptools for ATAC-seq and CUT&Tag-seq at four stages of HUVECs. **(B)** The fragment size distribution plot generated by ATAC-seq. **(C)** Box plots of ATAC signals after quantile normalization. Box plots are shown with the center (median), upper, and lower quartile range per sample, and thick lines indicate the median values. **(D)** Left: the mapping ratio of CUT&Tag-related genes to the human reference genome, right: Q30 scores of CUT&Tag. **(E)** Left: the mapping ratio of ATAC-seq-related genes to the human reference genome, right: Q30 score of ATAC-seq. **(F)** Left: the mapping ratio of RNA-seq reads to the human reference genome, right: Q30 score of RNA-seq.

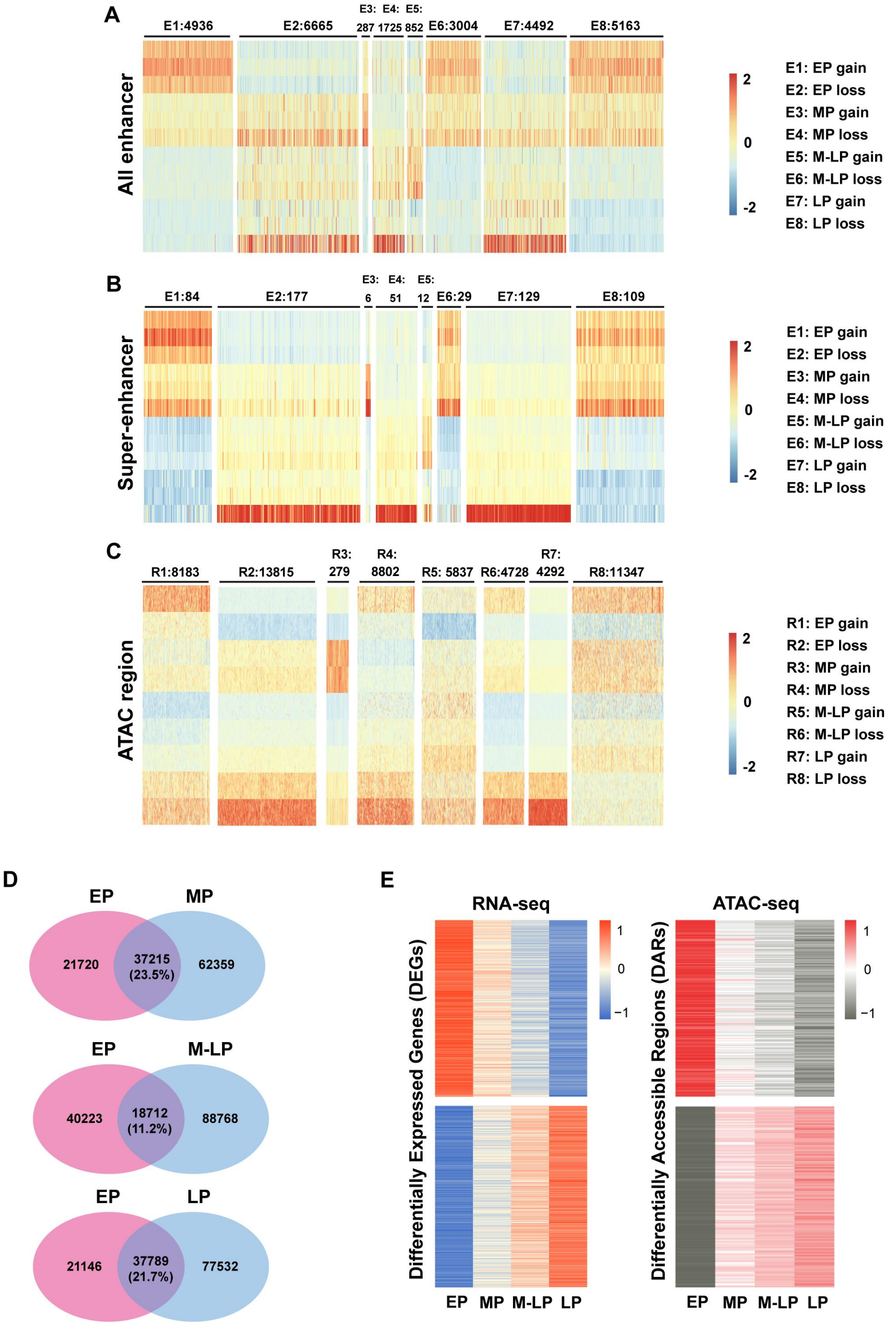

Figure S3. H3K27ac and ATAC landscapes define the active regulatory elements during endothelial cell senescence. (A-B) Heatmaps of the altered enhancers across four stages of HUVECs with H3K27ac enrichment signals. Eight groups (E1-E8) of all enhancers (A) or super-enhancers (B) with H3K27ac enrichment signals and the indicated number of enhancer elements are presented. **(C)** Heatmap of the altered accessible chromatin regions across four stages of HUVECs with ATAC-seq enrichment signals. **(D)** Venn diagrams illustrating the number of shared ATAC-seq peaks among MP, M-LP, LP, and EP, respectively. **(E)** Heatmap of DEGs from RNA-seq and DARs from ATAC-seq data at four stages of HUVECs.

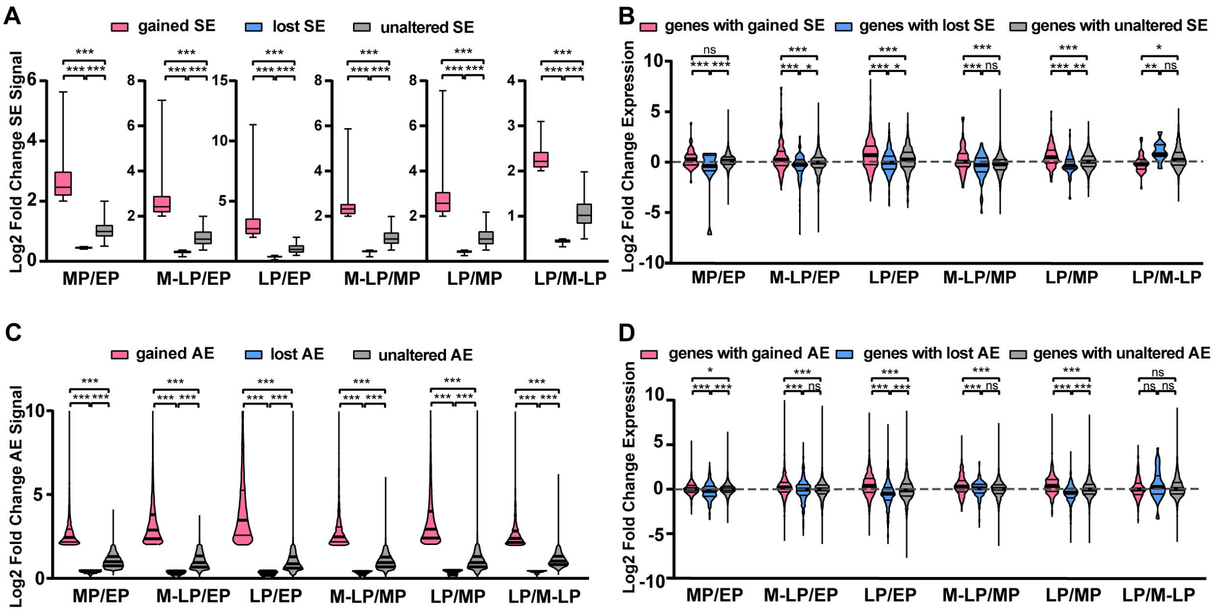

Figure S4. Differential enhancers associated with aberrant transcriptional programs. (A) Fold change of H3K27ac signals for super-enhancer. **(B)** The expression levels of differential SE-related genes. **(C)** Fold change of H3K27ac signals for all-enhancer. **(D)** The expression levels of differential AE-related genes. Unaltered SE or AE-related genes were used as controls. Box plots show the quartile spacing of the data, with bolded black lines indicating median values that extend to the lowest or highest data point. SE, super-enhancer, AE, all-enhancer. Data are presented as means ± SD, Student's *t*-test, $^{**}P < 0.01$, $^{***}P < 0.001$.

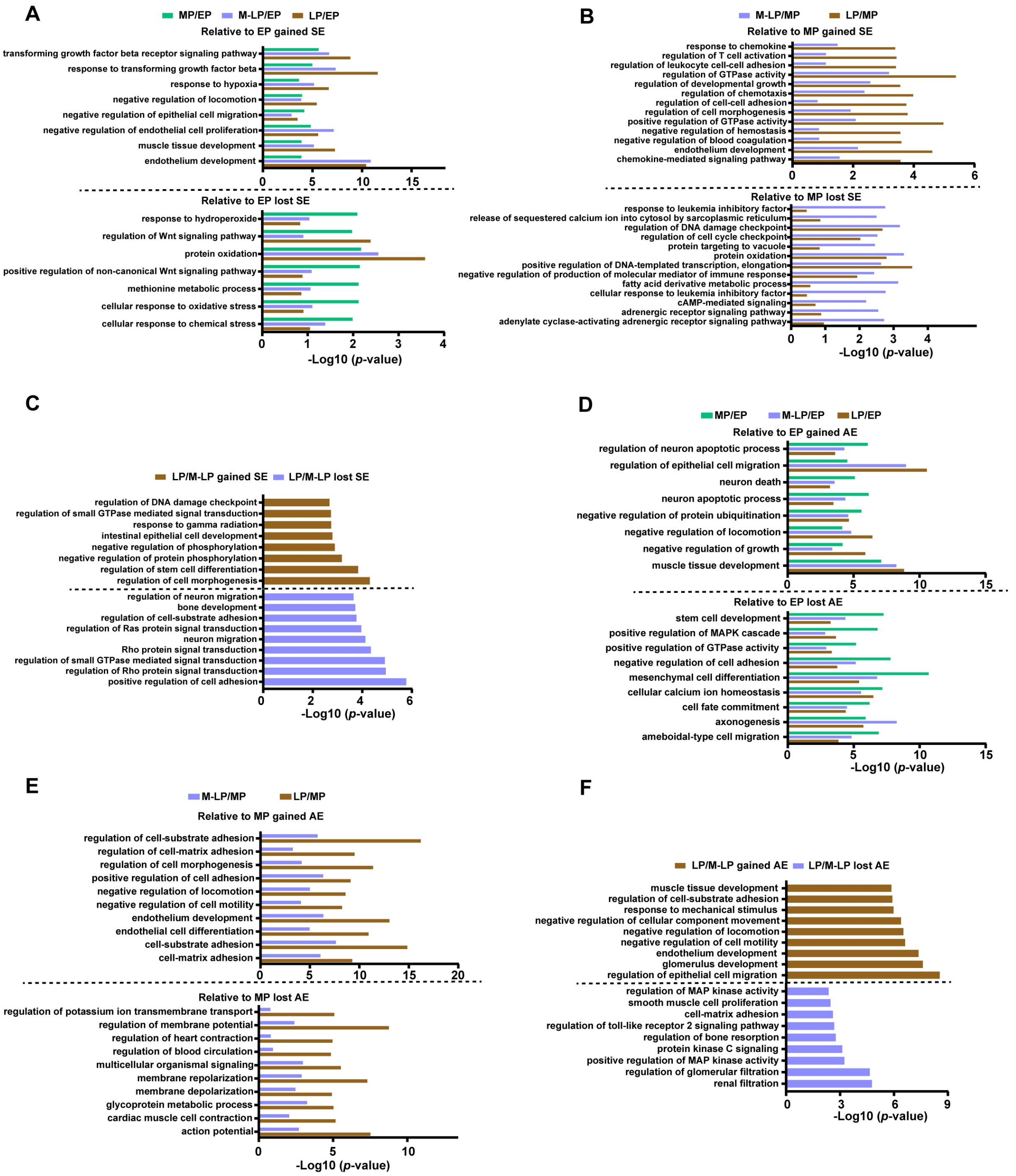

Figure S5. Gained SE-related genes of senescent stage are enriched in the basic senescence pathway. (A) Gene Ontology (GO) analysis of the gained or lost SE-related genes in the MP, M-LP, and LP compared to EP, respectively. **(B)** GO analysis of the gained or lost SE-related genes in the M-LP and LP compared to MP, respectively. **(C)** GO analysis of the gained or lost SE-related genes in the M-LP compared to LP. **(D)** GO analysis of the gained or lost AE-related genes in the MP, M-LP, and LP compared to EP, respectively. **(E)** GO analysis of the gained or lost AE-related genes in the M-LP and LP compared to MP, respectively. **(F)** GO analysis of the gained or lost AE-related genes in the M-LP compared to LP. SE, super-enhancer, AE, all-enhancer.

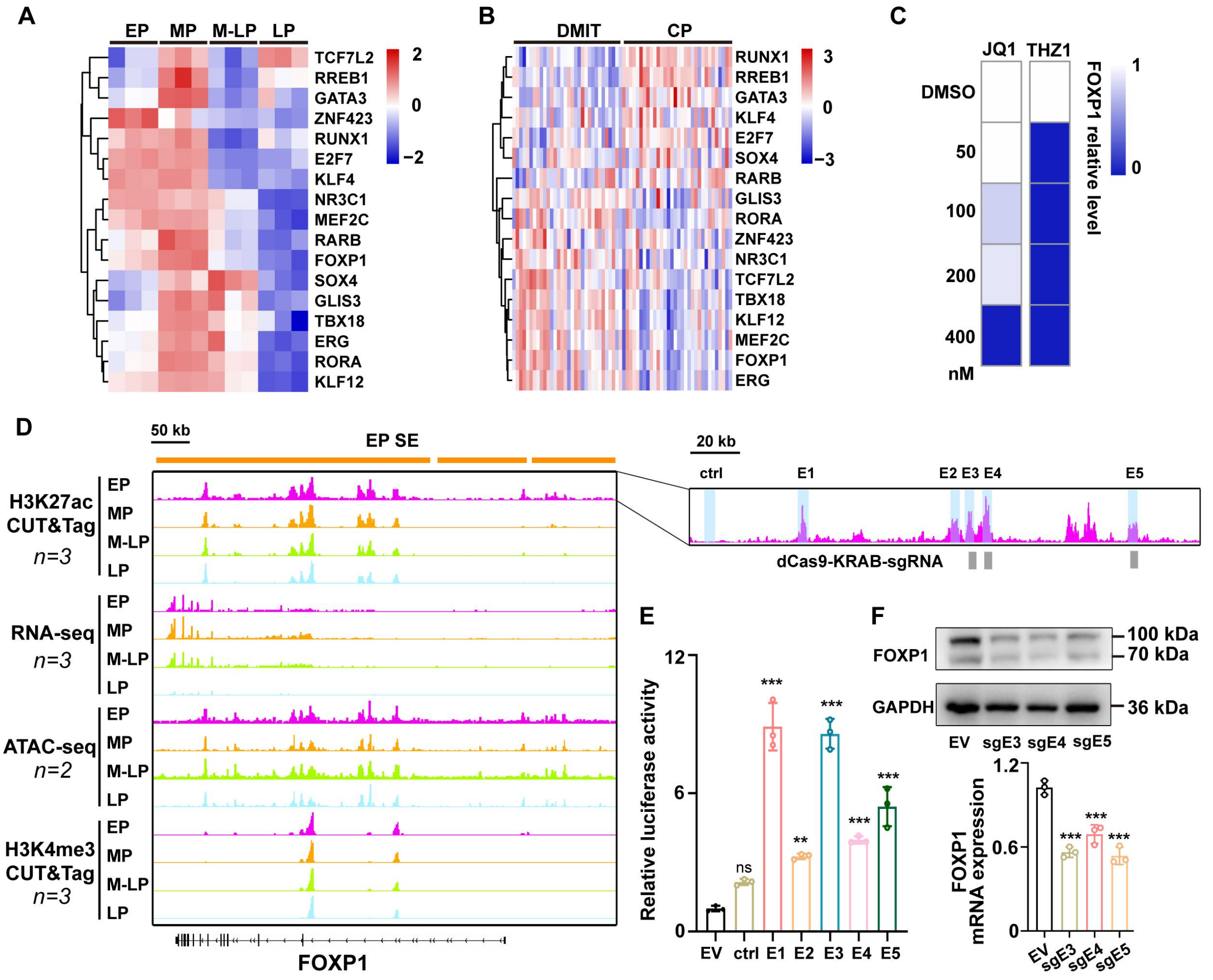

Figure S6. The core transcription factor FOXP1 is driven by super-enhancer. (A) The heatmap displays normalized gene expression across EP, MP, M-LP, and LP. The colors represent the normalized TPM values. **(B)** Heatmap of the expression of candidate core transcription factors in DMIT (distant macroscopically intact tissue) and CP (core of the plaque) samples from GEO database, GSE43292. The colors represent the normalized TPM values. **(C)** RT-qPCR analysis of FOXP1 expression following treatment with varying concentrations of THZ1 and JQ1 in HUVECs. **(D)** Viewable histone CUT&Tag signals, ATAC-seq, and RNA-seq at the *FOXP1* gene locus across four stages of HUVECs. **(E)** FOXP1 super-enhancer regions were selected within dense regions of H3K27ac binding peaks, encompassing an average of 2-3 kb of DNA sequence per segment. A negative control (ctrl) was included, along with a statistical graph depicting enhancer activity on the right. **(F)** Western blot and RT-qPCR analyses of FOXP1 expression following inhibition of the major enhancers' activity. Data are presented as means ± SD, one-way ANOVA, $^{**}P < 0.01$, $^{***}P < 0.001$.

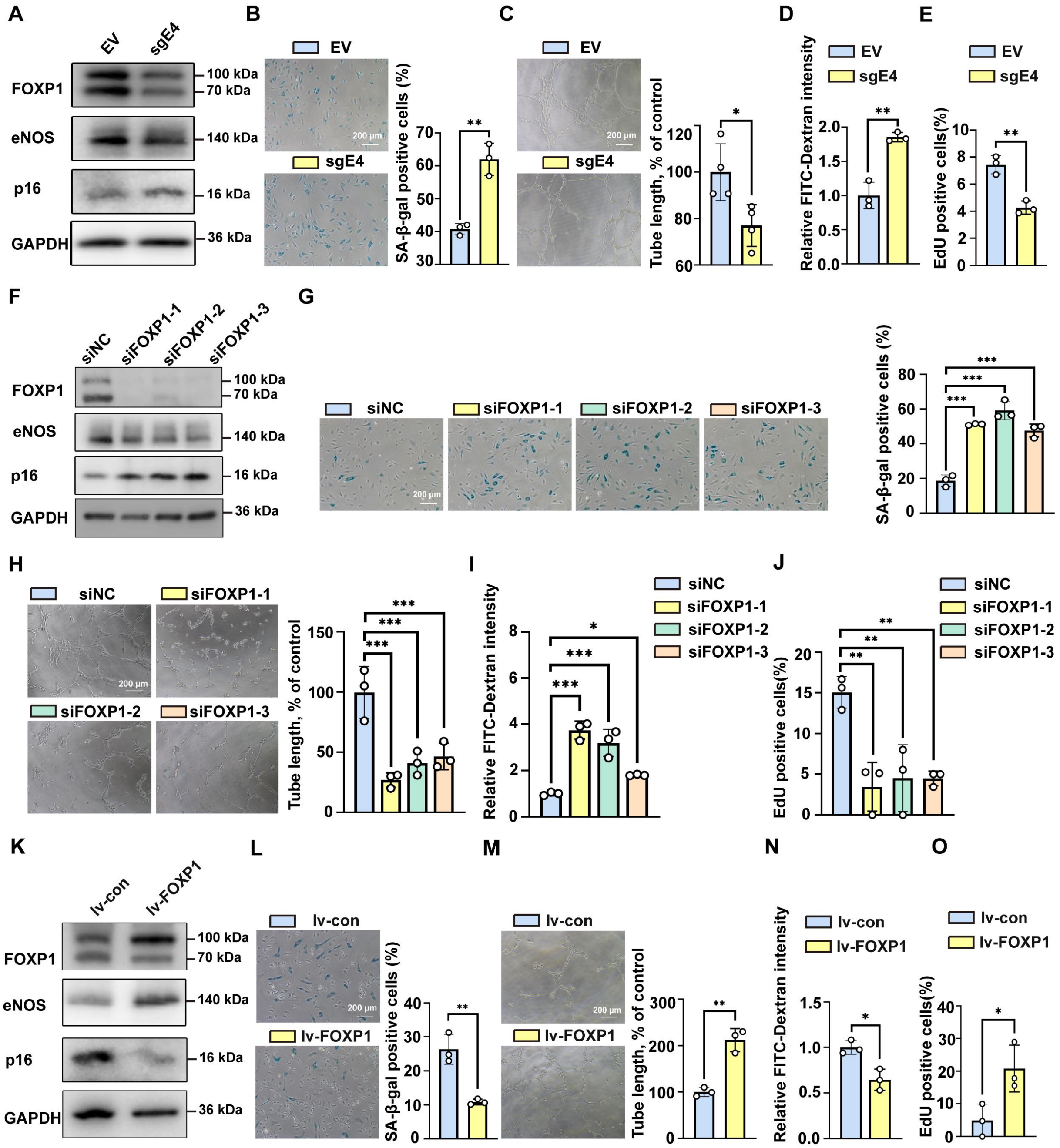

Figure S7. FOXP1 delays endothelial cell senescence and alleviates endothelial dysfunction. (A) Western blot analysis of FOXP1, eNOS, and p16 protein expression in HUVECs following the inhibition of E4 activity. **(B-C)** Representative images of SA-β-gal staining (B) and tubule formation (C) in HUVECs after the inhibition of E4 activity. **(D-E)** The experiments assessing endothelial cell permeability (D) and the proportion of proliferating cells (E) were conducted following the inhibition of E4 activity. **(F)** Western blot analysis of FOXP1, eNOS, and p16 protein expression in HUVECs following the knockdown of FOXP1. **(G-H)** Representative images of SA-β-gal staining (G) and tubule formation (H) by HUVECs after the knockdown of FOXP1. **(I-J)** The experiments assessing endothelial cell permeability (I) and the proportion of proliferating cells (J) were conducted following the

knockdown of FOXP1. **(K)** Western blot analysis of FOXP1, eNOS, and p16 protein expression in HUVECs following the overexpression of FOXP1. **(L-M)** Representative images of SA-β-gal staining (L) and tubule formation (M) by HUVECs after the overexpression of FOXP1. **(N-O)** The experiments assessing endothelial cell permeability (N) and the proportion of proliferating cells (O) were conducted following the overexpression of FOXP1. EV, empty vector, lv, lentivirus. Data are presented as the mean ± SD. In G-J, the statistical significance was calculated by one-way ANOVA. In B-E and L-O, the statistical significance was calculated by Student's *t*-test, **P* < 0.05, ***P* < 0.01, ****P* < 0.001.

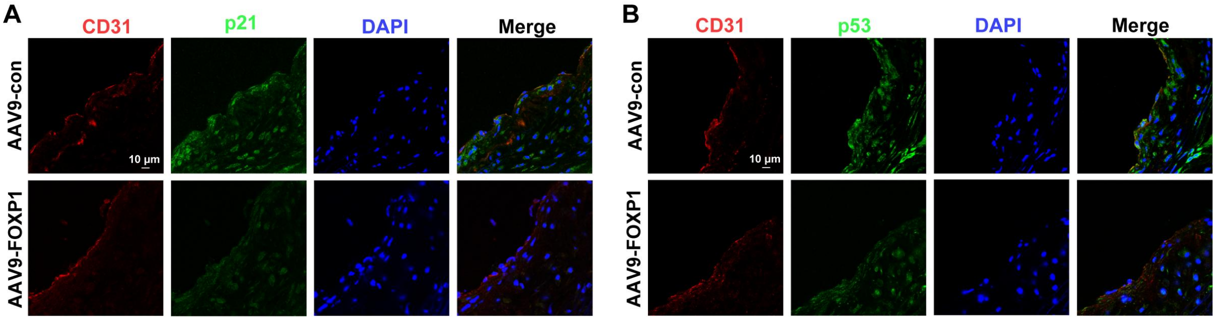

Figure S8. Endothelial cell-specific overexpression of FOXP1 can delay endothelial cell senescence. (A) Immunofluorescence staining of p21 and CD31 in the intima of ApoE KO mice following injection of AAV9-Con or AAV9-FOXP1. **(B)** Immunofluorescence staining of p53 and CD31 in the intima of ApoE KO mice following injection of AAV9-Con or AAV9-FOXP1.

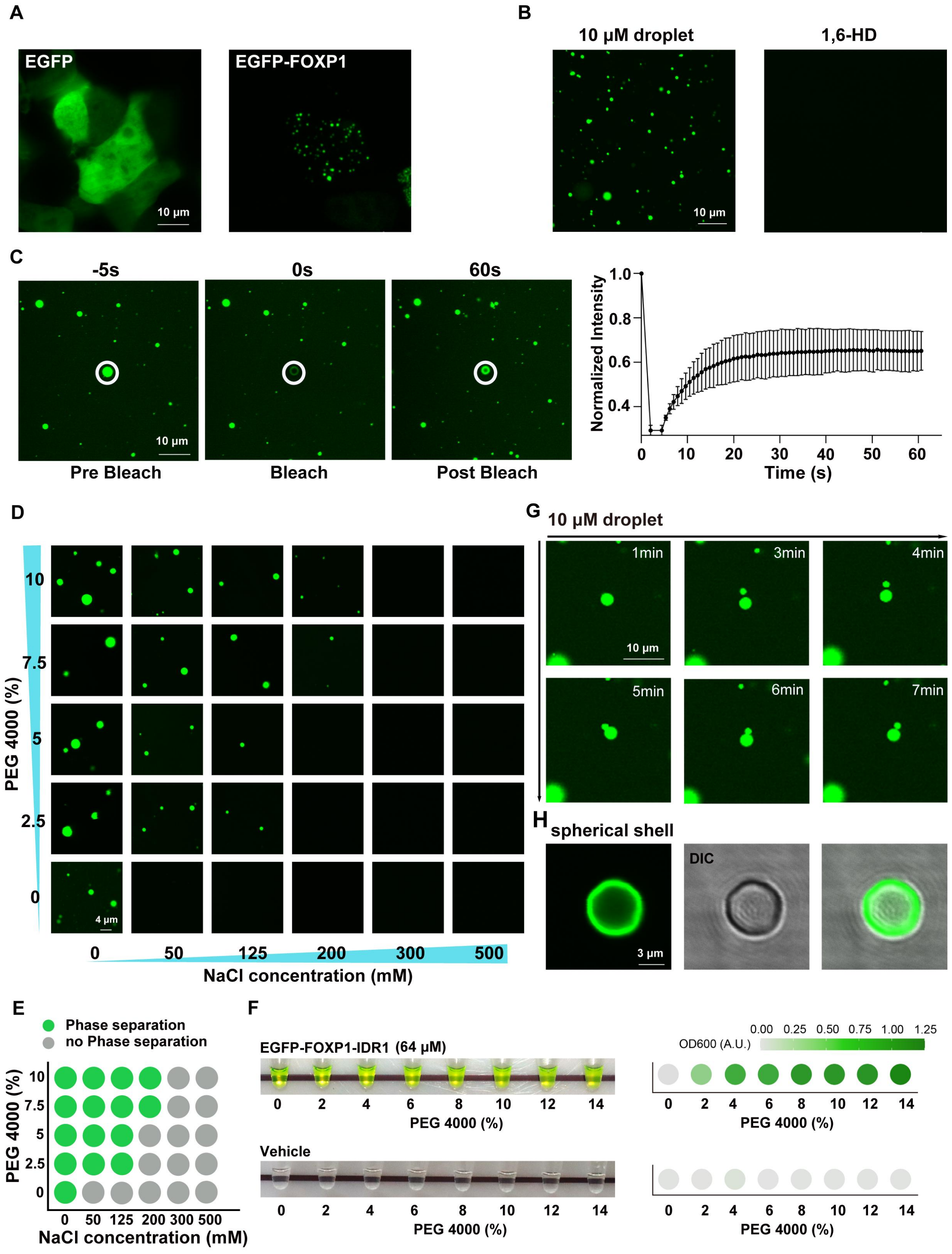

Figure S9. Physicochemical factors affecting phase separation of FOXP1. (A) Phase separation was observed after transient transfection of the pcDNA3.1-EGFP-FOXP1 plasmid in HEK293T cells. **(B)** The disruptive effect of 10% 1,6-hexanediol on EGFP-FOXP1-IDR1 protein droplets. **(C)** FRAP analysis of EGFP-FOXP1-IDR1 protein droplets. Time-lapse

images (left); quantification of fluorescence intensity during FRAP assay (right). **(D)** Images of the formation of EGFP-FOXP1-IDR1 proteins at different concentrations of PEG 4000 and NaCl. **(E)** Schematic illustration of the effect of D. **(F)** The impact of varying concentrations of PEG 4000 on the turbidity of EGFP-FOXP1-IDR1 protein, with turbidity measured as OD600. **(G)** Images depicting the spontaneous fusion of EGFP-FOXP1-IDR1 protein droplets. **(H)** EGFP-FOXP1-IDR1 protein droplets display a spherical, shell-like structure.

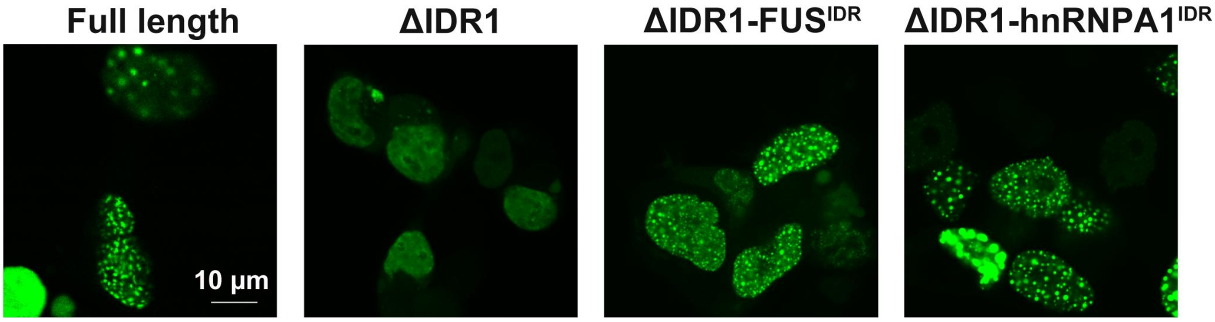

Figure S10. The fusion of IDR1-deficient variants with an IDR from FUS or hnRNPA1 fragments restores the LLPS.

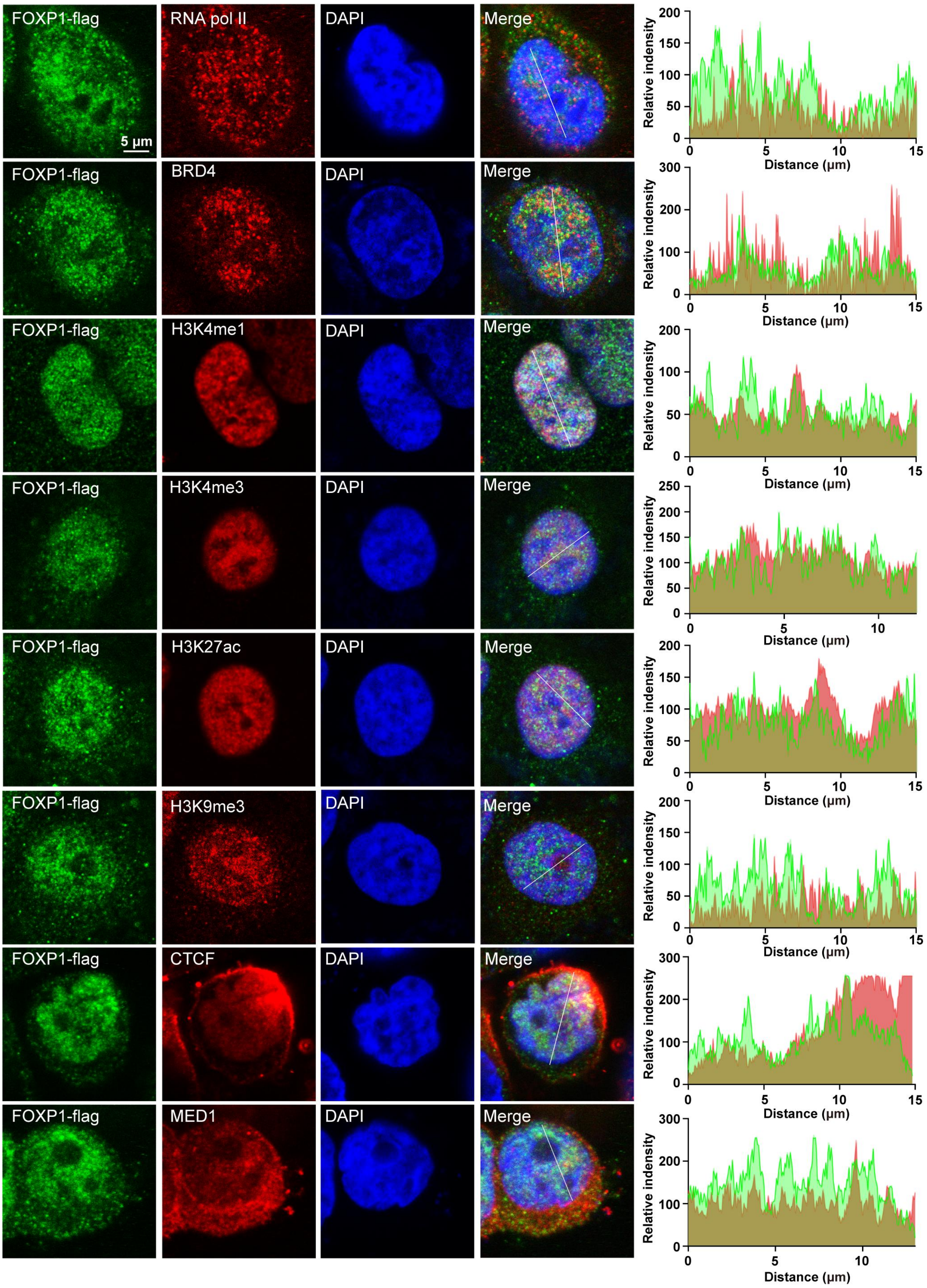

Figure S11. FOXP1 colocalizes with epigenetic factors. Immunofluorescence analysis was conducted to assess the colocalization of FOXP1 with other epigenetic factors. FOXP1 was expressed in fusion with flag tags, and colocalization analysis was performed along the white line using ImageJ.

Supplementary Movie 1. The EGFP-FOXP1 droplets underwent spontaneous fusion.

Movie 1.mp4

Supplementary Movie 2. Fluorescence recovery after photobleaching (FRAP) assays of the EGFP-FOXP1 droplets.

Movie 2.mp4

Supplementary Movie 3. The recombinant EGFP-FOXP1 protein underwent spontaneous fusion.

Movie 3.mp4

table S1 to S6

table S1. The list of peaks of sequencing data

table S2. The list of genes related to Figure 3A to D

table S3. The list of primer sequences

table S4. The list of sgRNA sequences

table S5. The list of siRNA sequences

table S6. The list of probe sequences for FISH
